# A Xylenol Orange-Based Screening Assay for the Substrate Specificity of Flavin-Dependent *para*-Phenol Oxidases

**DOI:** 10.3390/molecules23010164

**Published:** 2018-01-14

**Authors:** Tom A. Ewing, Aster van Noord, Caroline E. Paul, Willem J. H. van Berkel

**Affiliations:** 1 Laboratory of Biochemistry, Wageningen University & Research, Stippeneng 4, 6708 WE Wageningen, The Netherlands; tom.ewing@wur.nl (T.A.E.); aster.vannoord@wur.nl (A.v.N.); 2 Laboratory of Organic Chemistry, Wageningen University & Research, Stippeneng 4, 6708 WE Wageningen, The Netherlands; caroline.paul@wur.nl

**Keywords:** enzyme kinetics, flavoprotein, oxidase, screening assay, substrate specificity

## Abstract

Vanillyl alcohol oxidase (VAO) and eugenol oxidase (EUGO) are flavin-dependent enzymes that catalyse the oxidation of *para*-substituted phenols. This makes them potentially interesting biocatalysts for the conversion of lignin-derived aromatic monomers to value-added compounds. To facilitate their biocatalytic exploitation, it is important to develop methods by which variants of the enzymes can be rapidly screened for increased activity towards substrates of interest. Here, we present the development of a screening assay for the substrate specificity of *para*-phenol oxidases based on the detection of hydrogen peroxide using the ferric-xylenol orange complex method. The assay was used to screen the activity of VAO and EUGO towards a set of twenty-four potential substrates. This led to the identification of 4-cyclopentylphenol as a new substrate of VAO and EUGO and 4-cyclohexylphenol as a new substrate of VAO. Screening of a small library of VAO and EUGO active-site variants for alterations in their substrate specificity led to the identification of a VAO variant (T457Q) with increased activity towards vanillyl alcohol (4-hydroxy-3-methoxybenzyl alcohol) and a EUGO variant (V436I) with increased activity towards chavicol (4-allylphenol) and 4-cyclopentylphenol. This assay provides a quick and efficient method to screen the substrate specificity of *para*-phenol oxidases, facilitating the enzyme engineering of known *para-*phenol oxidases and the evaluation of the substrate specificity of novel *para*-phenol oxidases.

## 1. Introduction

Lignin, one of the major constituents of lignocellulosic biomass, is a heterogenous aromatic polymer, formed from the monolignols *para*-coumaryl alcohol, coniferyl alcohol and sinapyl alcohol through a radical coupling process [1,2]. It is obtained as a by-product during the processing of plant biomass in biorefineries and as such represents an attractive renewable feedstock of aromatic compounds. In order to harness the full potential of lignin as a renewable chemical feedstock, it is desirable to develop novel methods to depolymerise it to monomeric aromatic compounds and subsequently convert these to value-added compounds. One way in which lignin-derived aromatic molecules can be converted to value-added compounds is through the action of oxidative enzymes. Two such enzymes are the flavin-dependent oxidases vanillyl alcohol oxidase (VAO) from *Penicillium simplicissimum* and eugenol oxidase (EUGO) from *Rhodococcus jostii* RHA1 [3,4], which both belong to auxiliary activity family 4 (AA4) of the carbohydrate active enzyme (CAZy) database [5].

**Scheme 1:**
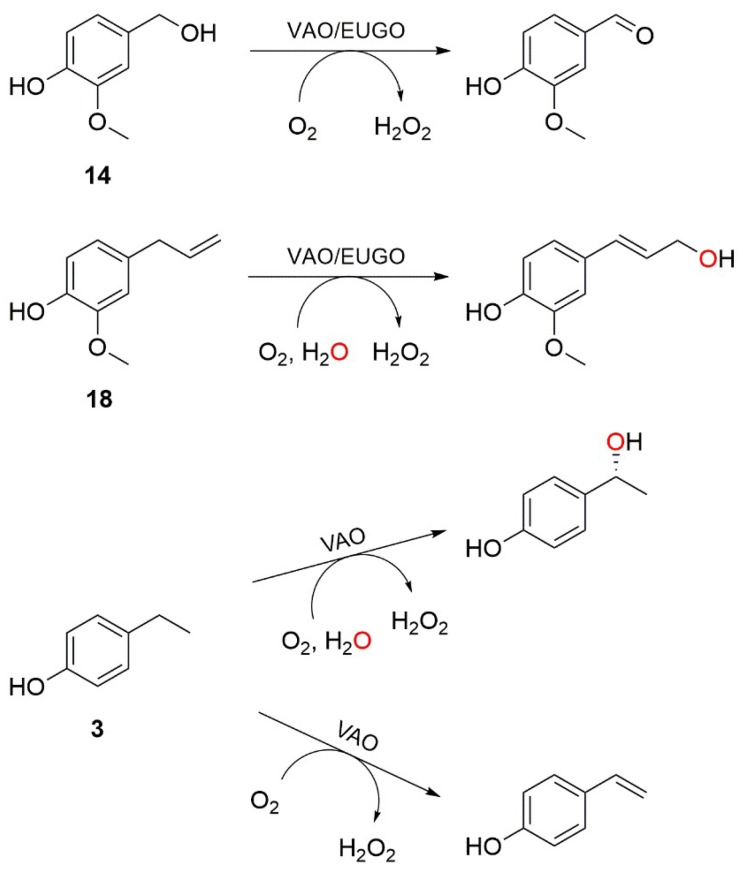
Examples of reactions catalysed by vanillyl alcohol oxidase (VAO) and/or eugenol oxidase (EUGO). Both enzymes can catalyse the oxidation of vanillyl alcohol (**14**) to the aldehyde vanillin and of eugenol (**18**) to coniferyl alcohol. VAO converts 4-ethylphenol (**3**) to a mixture of products consisting of 4-vinylphenol and 1-(4′-hydroxyphenyl)ethanol. The hydroxylation reaction occurs enantioselectively, predominantly yielding the (*R*)-enantiomer of the alcohol. In contrast, previous studies failed to reveal any activity towards **3** for EUGO [13].

VAO (EC 1.1.3.38) and EUGO (EC 1.1.3.x) are members of the VAO/PCMH flavoprotein family, which includes flavoenzymes characterised by the presence of a conserved FAD-binding domain [6,7]. VAO and EUGO both catalyse the two-electron oxidation of *para*-substituted phenols at the Cα position of their substituent (Scheme 1). Molecular oxygen acts as the electron acceptor for the reaction, being converted to hydrogen peroxide. Despite the fact that VAO and EUGO share significant sequence similarity (45% identical) and very similar secondary and tertiary structures, they differ in terms of their oligomerisation state and substrate specificity [8,9]. VAO displays a broad substrate specificity, catalysing the oxidation of alcohols to aldehydes, the oxidative deamination of amines, the oxidative demethylation of ethers, the dehydrogenation or hydroxylation of alkyl groups and the hydroxylation of allyl groups [10,11]. EUGO displays a somewhat narrower substrate specificity. Although alcohols and 4-allylphenols are good substrates for the enzyme, 4-alkylphenols and ethers are hardly accepted [4]. In terms of its oligomerisation state, VAO is predominantly octameric in solution, though active dimers may also be present under certain conditions [3,12]. In contrast, EUGO is exclusively dimeric in solution, with its structure strongly resembling that of a dimer of VAO [9].

A number of the reactions catalysed by VAO and EUGO are of interest for potential industrial applications. The oxidation of vanillyl alcohol (4-hydroxy-3-methoxybenzyl alcohol) or vanillyl amine [4-(aminomethyl)-2-methoxyphenol] yields vanillin (4-hydroxy-3-methoxybenzaldehyde), the main flavour and fragrance compound in vanilla [14]. VAO can also be used for the synthesis of chiral secondary alcohols, with the oxidation of short-chain linear 4-alkylphenols yielding the (*R*)-enantiomers of the corresponding alcohols in high enantiomeric excess [15]. The hydroxylation of 4-allylphenols by VAO or EUGO has been employed in multi-enzyme cascades for the synthesis of the plant lignan pinoresinol and model lignin compounds [16,17,18]. The hydroxylation of eugenol (4-allyl-2-methoxyphenol) by VAO has also garnered interest as the first step in fermentation processes for the conversion of eugenol to ferulic acid, which can be used as a precursor for vanillin [19,20,21,22,23,24].

Up to now, the molecular determinants of the differences in substrate specificity between VAO and EUGO are unclear. The differing oligomerisation states of the enzymes do not appear to be involved, as a VAO variant that exclusively forms dimers displayed similar catalytic properties to the wild-type enzyme [13]. To improve our understanding of the determinants of the substrate specificity of these *para-*phenol oxidases and to facilitate efforts to modify their reactivity by enzyme engineering, it would be beneficial to be able to rapidly screen variants of VAO and EUGO for activity towards multiple substrates. To enable this, we here describe a method for the rapid screening of the substrate specificity of *para*-phenol oxidases using the ferric-xylenol orange complex method for the detection of hydrogen peroxide.

The ferric-xylenol orange complex method (xylenol orange assay) is a colorimetric method for the determination of the concentration of hydroperoxides. When an assay solution containing Fe^2+^ ions and xylenol orange is added to a sample containing a hydroperoxide under acidic conditions, the hydroperoxide will oxidise Fe^2+^ to Fe^3+^. The Fe^3+^ subsequently forms a complex with xylenol orange that can be quantified from its absorbance at 560 nm [25,26]. The assay has been applied to measure the concentration of lipid or protein hydroperoxides in biological samples and as an activity assay for the lipid hydroperoxide-forming enzyme lipoxygenase [27,28,29]. Detection of hydrogen peroxide via the ferric-xylenol orange complex method can be used to detect the activity of oxidases and has been applied in a biosensor for lactose and in an in-gel screening assay for l-amino acid oxidase activity [30,31].

Methods used previously to determine the activity of VAO or EUGO typically relied on the measurement of the absorption of reaction products or the consumption of oxygen [4,10]. These assays are not suitable for high-throughput substrate specificity screening as they are either dependent on the identity of the phenolic reaction product (measuring absorption of the product) or cannot easily be performed for multiple samples simultaneously (measuring consumption of oxygen). In contrast, the xylenol orange assay does not depend on the identity of the phenolic reaction product and multiple samples can be evaluated simultaneously by performing the assay in a 96-wells plate. The xylenol orange assay is also better suited for our purpose than assays based on the detection of hydrogen peroxide via the formation of a coloured compound by peroxidases, as the phenolic substrates converted by VAO and EUGO are typically also substrates for peroxidases [32].

The method reported here consists of an efficient benchtop purification of His-tagged versions of VAO and EUGO, followed by a rapid screening of their activity towards a set of twenty-four potential substrates using the xylenol orange assay. After developing the procedure using the wild-type enzymes, we applied the assay to screen fourteen active-site variants for alterations in their substrate specificity. This led to the identification of new substrates of the wild-type enzymes and two variants that displayed increased activity towards at least one substrate.

## 2. Results

### 2.1. Purification and Characterisation of His-VAO and EUGO-His

To enable screening of the substrate specificity of multiple VAO and EUGO variants, we first needed a method for the rapid and efficient purification of the enzymes. To this end, we expressed the enzymes from new expression vectors containing genes encoding His-tagged variants of the enzymes. VAO was expressed as an N-terminally His-tagged protein, His-VAO, and EUGO was expressed as a C-terminally His-tagged protein, EUGO-His. To evaluate whether these constructs can be used for our study, we expressed and purified the His-tagged wild-type enzymes and evaluated whether their catalytic properties are similar to those of the non-His-tagged proteins.

Both His-tagged enzymes were successfully expressed in *E. coli* and purified by a simple benchtop procedure consisting of a single affinity chromatography step using a Ni-NTA column followed by buffer exchange using a desalting column. The purified enzymes contained flavin, as determined from their characteristic flavin absorption spectra (Figures S1 and S2). Upon precipitation of the proteins using trichloroacetic acid, a yellow pellet and colourless supernatant were obtained, demonstrating that the flavin cofactor is covalently bound to the protein. To determine whether the presence of a His-tag on the protein affects catalysis, we determined the steady-state kinetic parameters for the oxidation of vanillyl alcohol by His-VAO and EUGO-His (Table 1). This revealed that the catalytic properties of the His-tagged enzymes are highly similar to those of the non-His-tagged enzymes, demonstrating that the introduction of the His-tag does not affect catalysis. Therefore, these His-tagged variants of VAO and EUGO provide a suitable experimental system to rapidly purify variants of the enzymes and study their catalytic properties.

### 2.2. Development of the Xylenol Orange Assay Using His-VAO and EUGO-His

**Figure 1:**
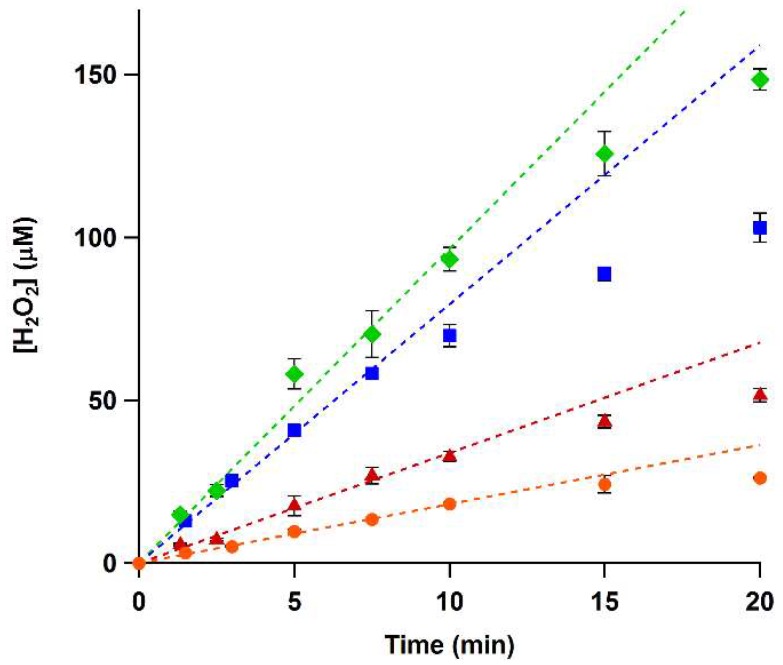
Conversion of eugenol or vanillyl alcohol by His-tagged vanillyl alcohol oxidase (His-VAO) or His-tagged eugenol oxidase (EUGO-His) followed in time using the xylenol orange assay. His-VAO ([●] for vanillyl alcohol, [♦] for eugenol) or EUGO-His ([■] for vanillyl alcohol, [▲] for eugenol) was incubated with 2 mM substrate in 50 mM potassium phosphate buffer, pH 7.5, at room temperature (19–20 °C). Reactions were allowed to proceed for the specified time, after which the amount of hydrogen peroxide formed was determined using the xylenol orange assay. Error bars represent the standard deviation of triplicate measurements. Broken lines are fits to the linear range of the data that were used to determine reaction rates (see Table 2).

To determine whether the xylenol orange assay can be used to accurately measure the activity of His-VAO and EUGO-His, we used it to follow the oxidation of vanillyl alcohol and eugenol by the enzymes in time. To this end, 2 mM vanillyl alcohol or eugenol was allowed to react with EUGO-His or His-VAO for time periods ranging from one to twenty min, after which the amount of hydrogen peroxide formed was determined using the xylenol orange assay. In all cases, the measured hydrogen peroxide concentration was found to increase in time. The increase was linear for the first ten min of the reactions, with the exception of the oxidation of vanillyl alcohol by EUGO-His, where the increase was linear for the first 7.5 min of the reaction (Figure 1).

Reaction rates were determined by fitting the linear range of the data. These rates (Table 2) were similar to the *k*_cat_ values determined previously using other methods, as would be expected considering that our reactions were performed using saturating substrate concentrations. Thus, determination of the amount of hydrogen peroxide formed using the xylenol orange assay is a suitable way to determine the rates of reactions catalysed by His-VAO or EUGO-His.

Subsequently, we used the xylenol orange assay to evaluate the conversion of a set of twenty-four (potential) substrates by wild-type His-VAO and EUGO-His. The set of compounds contained various 4-(cyclo)alkylphenols, 4-hydroxybenzyl alcohols, 4-allylphenols and a 4-hydroxybenzylic amine. In addition to these *para*-substituted phenols, 2-hydroxy- and 3-hydroxybenzyl alcohol were included in the set of compounds in order to screen for variants that no longer have a strict specificity for *para*-substituted phenols. For a full list of the compounds and their structural formulae, see Table 3.

**Figure 2:**
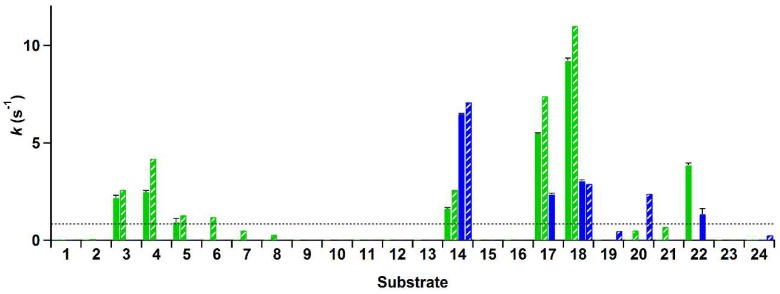
Rate of the reactions of His-tagged vanillyl alcohol oxidase (His-VAO) and His-tagged eugenol oxidase (EUGO-His) with the compounds used in the substrate specificity screening in 50 mM potassium phosphate buffer, pH 7.5, at room temperature (19–20 °C) as determined using the xylenol orange assay. Solid bars give the reaction rates measured with the xylenol orange assay for His-VAO (green) and EUGO-His (blue). Error bars represent the standard deviation of duplicate measurements. Striped bars give *k*_cat_ values measured previously by other methods with the non-His-tagged enzymes. These *k*_cat_ values were measured under identical experimental conditions, with the exception of the temperature, which was 25 °C instead of 19–20 °C. The striped horizontal line indicates the detection limit of the xylenol orange assay, which corresponds to a reaction rate of 0.83 s^−1^. The *k*_cat_ values were from the following sources. VAO: compounds **1**, **4** [33], **5**–**8**, **20**, **21** [11], **2**, **24** [14], **3**, **14**, **18** [13], **17** [34]. EUGO: **20**, **24** [4], **19** [9], **14**, **18** [13]. Numbering is according to Table 3.

The rate of conversion of these compounds by His-VAO and EUGO-His at a substrate concentration of 2 mM was determined by allowing the enzymes to react with the substrate for ten min and measuring the amount of hydrogen peroxide formed using the xylenol orange assay. Rates of the reactions of His-VAO and EUGO-His with the tested substrates, calculated by assuming that the formation of hydrogen peroxide in time is linear during the reaction period, are shown in Figure 2. For substrates where a *k*_cat_ value has been determined for the non-His-tagged enzymes under similar experimental conditions, this value is shown for comparison. 

The reaction rates measured using the xylenol orange assay are generally in good agreement with previously obtained data regarding the substrate specificity of VAO and EUGO. For compounds for which the *k*_cat_ had previously been determined and found to lie above the detection limit, the reaction rates measured with the xylenol orange assay typically lie close to the *k*_cat_ values. This is to be expected, as the substrate concentration used for the xylenol orange assay (2 mM) is at least ten times the *K*_m_ for all these compounds and as such the measurements were performed under saturating substrate concentrations. The only cases where no activity was observed with the xylenol orange assay despite the *k*_cat_ lying above the detection limit were the oxidation of 4-*n*-butylphenol (**6**) by His-VAO and 5-indanol (**20**) by EUGO-His. The *k*_cat_ for the oxidation of 4-*n*-butylphenol by VAO (1.2 s^−1^) is only slightly higher than the detection limit of the assay (0.83 s^−1^) and therefore it is not too surprising that no activity was detected for its oxidation using the xylenol orange assay. However, the *k*_cat_ for the oxidation of 5-indanol by EUGO (2.4 s^−1^) lies well above the detection limit of the xylenol orange assay. To investigate this discrepancy further, the rate of oxidation of 2 mM 5-indanol by EUGO-His was determined by monitoring the consumption of oxygen during the reaction. The rate of oxygen consumption was found to be 0.064 s^−1^, significantly lower than the detection limit for the xylenol orange assay. This is in reasonable agreement with the rate of oxidation of 2 mM 5-indanol by EUGO determined by Nguyen et al. (0.18 s^−1^; the higher activity may be attributable to the addition of 10% DMSO as a co-solvent). Therefore, at a substrate concentration of 2 mM, the rate of conversion of 5-indanol by EUGO-His is significantly lower than the detection limit of the xylenol orange assay. For all substrates where the previously determined *k*_cat_ value is lower than the detection limit, no activity was observed using the xylenol orange assay.

**Scheme 2:**
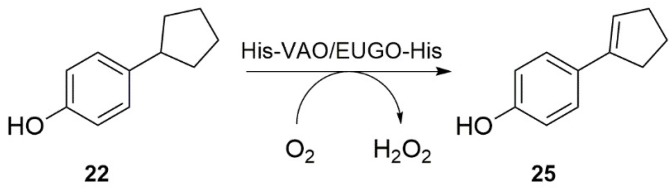
Oxidation of 4-cyclopentylphenol (**22**) to 4-(1-cyclopenten-1-yl)phenol (**25**) by His-tagged vanillyl alcohol oxidase (His-VAO) or His-tagged eugenol oxidase (EUGO-His), as catalysed in 50 mM potassium phosphate buffer, pH 7.5, at 25 °C.

Interestingly, our substrate specificity screening revealed that a number of compounds not previously described as substrates of VAO or EUGO are converted by the enzymes. Chavicol (**17**), which had previously been shown to be a substrate for VAO, was also converted by EUGO-His, though at a lower rate than by His-VAO. More surprisingly, both His-VAO and EUGO-His were active with 4-cyclopentylphenol (**22**), which had not previously been shown to be a substrate for either enzyme. Particularly with His-VAO, 4-cyclopentylphenol was quite efficiently converted, with the observed rate of 3.9 s^−1^ being higher than that observed for all other substrates except eugenol and chavicol. The ability of His-VAO and EUGO-His to convert 4-cyclopentylphenol was confirmed by allowing 1 μM His-VAO or EUGO-His to react with 2 mM 4-cyclopentylphenol at 25 °C and analysing the reaction products by GC and GC/MS (Figures S3 and S4). With both enzymes, the substrate was almost completely converted after two h (>99% conversion). The reaction yielded a single product, which was identified as 4-(1-cyclopenten-1-yl)phenol by GC/MS and ^1^H-NMR (**25**, Scheme 2, Figures S4 and S5). The rate of oxidation of 2 mM 4-cyclopentylphenol by His-VAO and EUGO-His was also determined by monitoring oxygen consumption during the reaction. This yielded rates of 3.7 s^−1^ for His-VAO and 1.7 s^−1^ for EUGO-His, in good agreement with the rates determined using the xylenol orange assay (3.9 s^−1^ and 1.3 s^−1^ for His-VAO and EUGO-His, respectively).

Taken together, these results demonstrate that the xylenol orange assay can be used to accurately measure the activity of His-VAO and EUGO-His towards a range of substrates and to identify hitherto unknown substrates of the enzymes. Therefore, it is a suitable assay for use in screening the substrate specificity of His-VAO and EUGO-His variants. Having established this, we set out to use the assay to screen a small library of His-VAO and EUGO-His variants for changes in their substrate specificity.

### 2.3. Analysis of the Substrate-Binding Pockets of VAO and EUGO

**Figure 3:**
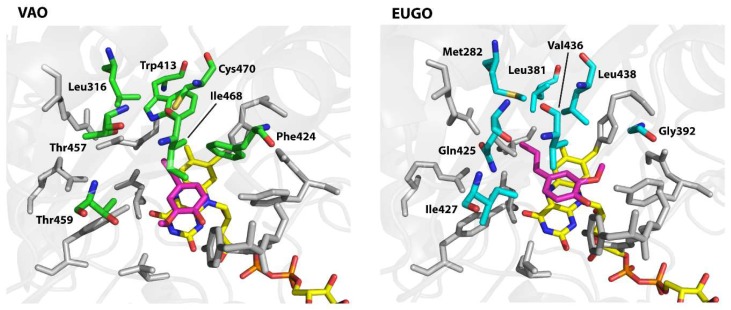
Structures of the substrate-binding pockets of vanillyl alcohol oxidase (VAO) and eugenol oxidase (EUGO) with the competitive inhibitor isoeugenol bound. Structures are shown in stick representation with residues that are identical in each protein shown in grey, residues that are different in each protein shown with green (VAO) or cyan (EUGO) carbon atoms, the FAD cofactor shown with yellow carbon atoms and isoeugenol shown with magenta carbon atoms. The VAO and EUGO structures were downloaded from the PDB website (PDB identifiers: 2VAO [8] and 5FXD [9] respectively). Figure was prepared using PyMOL v. 1.3 (Schrödinger LLC, New York, NY, USA).

To design the variants, we examined the active sites of the proteins to identify residues that differ between them and therefore may be responsible for the observed differences in substrate specificity. Both enzymes contain a solvent-inaccessible substrate-binding pocket on the *si*-side of the FAD cofactor. This pocket is lined by 19 residues of which 12 are identical in both enzymes (Figure 3). The conserved residues include all those known to be directly involved in catalysis, including Arg-504 (numbering of amino acids is as in non-His-tagged VAO), which is thought to stabilise the negative charge that develops at the N1–C2=O2 locus of FAD upon its reduction, Tyr-108 and Tyr-503, which stabilise the deprotonated form of phenolic substrates in the active site, Asp-170, which promotes flavin reduction by stabilising the reduced FAD cofactor through hydrogen bonding with the protonated N5 atom, and His-422, to which the FAD cofactor is covalently bound [8,34,35]. The seven differing residues form a cluster on the side of the substrate opposite the flavin cofactor. Interestingly, the competitive inhibitor isoeugenol binds in a different orientation in each enzyme. In EUGO, it is flipped 180° as compared to in VAO. This is likely due to the presence of Gly-392 in EUGO instead of the bulky Phe-424 found at this position in VAO. Based on these observations, we hypothesised that differences in the cluster of differing residues may be responsible for the observed differences in substrate specificity between VAO and EUGO. To investigate this, we made seven His-VAO and seven EUGO-His variants, exchanging each of the differing residues for the amino acid that is found at this position in the other enzyme and set out to study their activity and substrate specificity using the xylenol orange assay (for a list of the residues and variants see Table 4, numbering as in the sequences of non-His-tagged VAO and EUGO).

### 2.4. Substrate Specificity Screening of His-VAO and EUGO-His Variants

**Figure 4:**
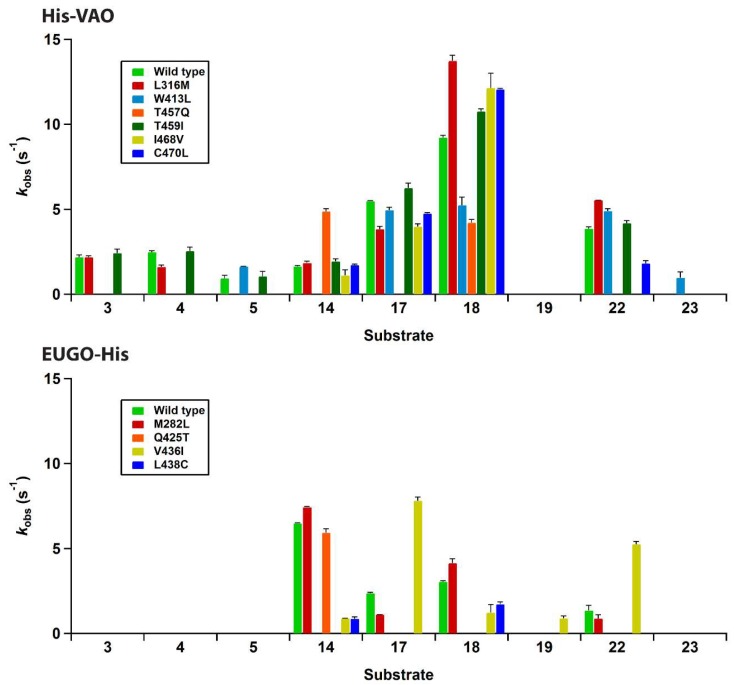
Rates of the reactions of His-tagged vanillyl alcohol oxidase (His-VAO) and His-tagged eugenol oxidase (EUGO-His) variants with the compounds used in the substrate specificity screening in 50 mM potassium phosphate buffer, pH 7.5, at room temperature (19-20 °C) as determined using the xylenol orange assay. Error bars represent the standard deviations of duplicate measurements. For all compounds that are not included in the graphs, no variants gave a signal above the detection limit of the assay. The F424G His-VAO variant was not studied, as it did not contain any flavin cofactor. For the L381W, G392F and I427T EUGO-His variants, no response higher than the detection limit was measured for any compound.

All fourteen variants were successfully expressed in *E. coli* and purified as described for the wild-type enzymes. A single variant, F424G His-VAO, did not contain any flavin after purification, as judged from the lack of a yellow colour and of the distinctive flavin absorption spectrum. Attempts to incorporate flavin into this variant by incubating it with FAD failed and it was not studied further. The other six His-VAO variants and all seven EUGO-His variants contained flavin as determined from their characteristic flavin absorption spectra (Figures S1 and S2). Upon precipitation of the proteins using trichloroacetic acid, a yellow pellet and colourless supernatant were obtained, demonstrating that the flavin cofactor is covalently bound to the protein. The absorption spectra of all seven EUGO-His variants had a similar shape to that of wild-type EUGO-His. A number of the His-VAO variants, most notably T457Q and C470L, displayed a somewhat altered flavin absorption spectrum, suggesting that in these variants the electronic environment of the flavin is slightly altered by the introduced mutations. The variants were screened for changes in their substrate specificity using the xylenol orange assay. The results of this screening assay are shown in Figure 4.

For His-VAO, all six remaining variants displayed activity with at least some of the tested compounds. Two of the variants, L316M and T459I, displayed substrate specificity profiles that were similar to that of the wild-type enzyme. The I468V and C470L variants displayed similar activity to the wild-type enzyme with the substrates vanillyl alcohol (**14**), chavicol (**17**) and eugenol (**18**). However, these variants did not display any activity towards linear 4-alkylphenols. The T457Q variant was the only variant to have significantly increased activity with vanillyl alcohol as a substrate, displaying a reaction rate that was approximately threefold higher than that of the wild-type enzyme. However, with all other substrates the T457Q variant displayed a decrease in activity. The only variant for which activity was detected with a compound for which no activity was detected for wild-type His-VAO was W413L. This variant displayed activity towards 4-cyclohexylphenol (**23**), a compound that so far had not been described as a substrate for VAO or EUGO. In addition to this novel reactivity, the W413L variant displayed activity towards 4-cyclopentylphenol (**22**), eugenol, chavicol and 4-isopropylphenol (**5**). No activity was detected with linear 4-alkylphenols or vanillyl alcohol.

To confirm that W413L His-VAO oxidises 4-cyclohexylphenol, 1 μM enzyme was incubated with 2 mM 4-cyclohexylphenol for 4 h at 25 °C and the reaction products were analysed by GC and GC/MS (Figures S6 and S7). This revealed that 4-cyclohexylphenol is indeed converted by the enzyme yielding a single product, though the degree of conversion was only 27%. GC/MS analysis revealed that the product displays a molecular ion at *m/z* 174.1, suggesting that it is formed by the dehydrogenation of the substrate. As VAO typically dehydrogenates the Cα-Cβ bond of alkyl groups, the product of 4-cyclohexylphenol oxidation is presumably 4-(1-cyclohexen-1-yl)phenol. Similar experiments were performed using wild-type His-VAO and EUGO-His to evaluate whether they are also capable of converting 4-cyclohexylphenol. This revealed that after a 4 h incubation period, wild-type His-VAO had converted 51% of the substrate yielding the same product as observed with W413L His-VAO. In contrast, no conversion of 4-cyclohexylphenol by wild-type EUGO-His was observed under these reaction conditions.

The substrate specificity screening of the EUGO-His variants revealed that three of the seven variants, L381W, G392F and I427T, did not display measurable activity towards any of the tested compounds. One of the four remaining variants, M282L, displayed a substrate specificity profile that was similar to that of wild-type EUGO-His. Another variant, Q425T, displayed similar activity to the wild-type enzyme with vanillyl alcohol, but did not display measurable activity towards any other compounds. The L438C variant displayed lowered activity towards all substrates as compared to the wild-type enzyme. The V436I variant displayed lowered activity towards vanillyl alcohol and eugenol. However, this variant had significantly increased activity towards chavicol and 4-cyclopentylphenol and also displayed activity towards the dimethoxylated substrate 2,6-dimethoxy-4-allylphenol (**19**). This compound had previously been described to be a substrate for EUGO [9], but the *k*_cat_ value for its oxidation (0.49 s^−1^) is lower than the detection limit of our assay, explaining the lack of activity detected for wild-type EUGO-His. No variants were identified that displayed activity towards compounds that had previously been identified as substrates for VAO, but not for EUGO, such as linear 4-alkylphenols.

In summary, the majority of the studied variants displayed similar or significantly reduced activity towards all tested compounds when compared to the corresponding wild-type enzyme. Two variants were identified that had significantly (more than twofold) increased activity towards one or more substrates.

## 3. Discussion

Here, we describe the development of a method for the efficient screening of variants of VAO and EUGO for alterations in their substrate specificities. The method is based on a facile benchtop purification of His-tagged versions of the enzymes, followed by a substrate specificity screening using a xylenol orange assay. After demonstrating that this method allows accurate determination of reaction rates of the wild-type enzymes with a range of substrates, we used it to screen a library of fourteen His-VAO or EUGO-His variants towards a set of twenty-four (potential) substrates. The use of the xylenol orange assay, a spectrophotometric detection method for the oxidase product hydrogen peroxide, allows the simultaneous measurement of activity towards a range of substrates in a high-throughput fashion. This compares favourably to previous methods for measuring the activity of VAO and EUGO. These either relied on measuring the absorption of the product of the reaction, making the detection method dependent on the substrate used and on prior knowledge of the identity of the product, or on measuring the consumption of oxygen, which is not practical for high-throughput screening purposes. Although generic methods for the high-throughput screening of oxidase activity have been developed in the past, they typically rely on peroxidase-coupled activity assays, where a peroxidase uses the formed hydrogen peroxide to catalyse the production of a coloured product [36]. This type of assay is not suitable for use with VAO or EUGO, as their phenolic substrates are typically also converted by peroxidases [32].

Although the number of substrates and variants used in our screen is relatively small, the xylenol orange assay is highly scalable, particularly when robotic pipetting systems are available. In our case, the limiting factor for the number of variants that can be screened is the protein purification method, which relies on the use of gravity-flow Ni-NTA columns. However, we did demonstrate that this single purification step allowed us to obtain sufficiently pure His-VAO and EUGO-His variants. High-throughput methods for the production and purification of His-tagged proteins using *E. coli* expression systems have been described [37]. Such a system could be used to achieve the purification of a large number of His-VAO or EUGO-His variants for use in the substrate specificity screening. Alternatively, cell free extracts of *E. coli* expressing the variants could be used for the assay. However, in our hands this gave poor results, most likely due to degradation of the formed hydrogen peroxide by catalase present in the *E. coli* extracts.

The substrate specificity profiling of wild-type His-VAO and EUGO-His led to the identification of 4-cyclopentylphenol as a substrate of the enzymes. Its conversion yielded 4-(1-cyclopenten-1-yl)phenol as the sole product. This is in agreement with the previous finding that while VAO can catalyse both the hydroxylation and the dehydrogenation of 4-alkylphenols, the dehydrogenation reaction is preferred when the alkyl side chain is relatively large [11]. Our results also revealed that His-VAO and the W413L His-VAO variant catalyse the dehydrogenation of 4-cyclohexylphenol, presumably to 4-(1-cyclohexen-1-yl)phenol. However, this reaction was not catalysed by EUGO-His.

Although the substrate specificity profiling of fourteen His-VAO or EUGO-His variants did not lead us to a thorough understanding of the molecular determinants of the differing substrate specificities of the enzymes, it did give some interesting insights into the importance of certain non-catalytic active site residues for enzyme activity. One interesting finding was that the F424G His-VAO variant did not contain any FAD when purified from *E. coli* and attempts to incorporate FAD into the protein failed. Possibly, the extra flexibility conferred by the introduction of a glycine residue in this variant destabilises the structure of the active site, leading to impaired flavin binding. Three of the seven EUGO-His variants, L381W, G392F and I427T, did not display any activity in the substrate specificity profiling, despite containing covalently bound flavin. Presumably, the introduced mutations affect the structure of the active site in such a way that the substrate is no longer bound in an orientation that is productive for catalysis.

The I468V and C470L His-VAO variants also displayed interesting changes in substrate specificity, maintaining similar reaction rates to the wild-type enzyme with vanillyl alcohol, chavicol and eugenol, but displaying no activity towards linear 4-alkylphenols. Ile-468 and Cys-470 are positioned close together at the top of the substrate-binding pocket (Figure 3). Possibly, the identity of these two residues is important for maintaining VAO’s reactivity with linear 4-alkylphenols. As the ability to convert linear 4-alkylphenols is one of the main differences between VAO and EUGO in terms of substrate specificity, it would be interesting to investigate whether further mutagenesis at these positions could create a EUGO variant that is capable of catalysing this reaction.

Only two variants that displayed significantly increased activity towards a compound for which activity was also detected with the wild-type enzymes were identified. T457Q His-VAO had a threefold increased reaction rate for the oxidation of vanillyl alcohol compared to wild-type His-VAO and I436V EUGO-His had three- and fourfold increased reaction rates for the oxidation of chavicol and 4-cyclopentylphenol respectively when compared to wild-type EUGO-His. It also displayed increased activity towards 4-allyl-2,6-dimethoxyphenol, though the magnitude of the increase could not be quantified as the reaction rate of wild-type EUGO-His with this compound is below the detection limit of the assay.

In summary, we developed an efficient method for screening variants of VAO and EUGO for changes in their substrate specificity consisting of the benchtop purification of His-tagged versions of the enzymes followed by substrate specificity screening using a xylenol orange assay. This method was employed for the substrate specificity profiling of the wild-type enzymes and of fourteen enzyme variants, leading to the identification of 4-cyclopentylphenol as a new substrate for both wild-type enzymes and of 4-cyclohexylphenol as a new substrate for wild-type His-VAO. Two variants, T457Q His-VAO and V436I EUGO-His were found to display significant increases in activity towards certain substrates. This method has the potential to be used to screen larger variant libraries in future, facilitating efforts to design VAO and EUGO variants for biocatalytic applications.

## 4. Materials and Methods

### 4.1. Materials

4-Hydroxy-3,5-dimethoxybenzyl alcohol was from Alfa Aesar (Haverhill, MA, USA). 4-*n*-Butylphenol and 4-*n*-nonylphenol were from Lancaster Synthesis (Haverhill, MA, USA). Ferrous sulphate heptahydrate was from Merck (Burlington, MA, USA). 4-Allylphenol (chavicol) was from Quest International (Naarden, The Netherlands). 4-Allylphenol-2,6-dimethoxyphenol, 4-allyl-2-methoxyphenol (eugenol), 4-(aminomethyl)-2-methoxyphenol hydrochloride (vanillyl amine), 4-*sec*-butylphenol, 4-cyclohexylphenol, 4-cyclopentylphenol, 4-ethylphenol, 4-*n*-hexylphenol, 2-hydroxy-benzyl alcohol, 3-hydroxybenzyl alcohol, 4-hydroxybenzyl alcohol, 4-hydroxy-2-methoxybenzyl alcohol, 4-hydroxy-3-methoxybenzyl alcohol (vanillyl alcohol), 5-indanol, 4-isopropylphenol, 2-methoxy-4-methylphenol (*para*-creosol), 4-methylphenol (*para*-cresol), 4-*n*-pentylphenol, 4-*n*-propylphenol, 5,6,7,8-tetrahydronaphthol and xylenol orange tetrasodium salt were from Sigma-Aldrich (St. Louis, MO, USA). All other chemicals were from commercial sources and of the purest grade available. The pJ404-His-VAO and pBAD-EUGO-His plasmids were a kind gift from Prof. Dr. Marco Fraaije (University of Groningen).

### 4.2. Site-Directed Mutagenesis

Plasmids encoding for the His-VAO and EUGO-His variants were created by linear whole-plasmid amplification from the pJ404-His-VAO and pBAD-EUGO-His plasmids respectively. The pJ404-His-VAO plasmid contains a version of the *vaoA* gene from *P. simplicissimum* that is codon-optimised for expression in *E. coli* behind the IPTG-inducible T5 promoter. The gene is extended with a sequence that encodes an N-terminal 6x-His-tag followed by a single glycine residue. The pBAD-EUGO-His plasmid contains the *eugo* gene from *Rhodococcus jostii* RHA1 with an extension that encodes the polypeptide sequence GKLGPEQKLISEEDLNSAVDHHHHHH at the C-terminus of the protein. The extension contains both a C-terminal 6x-His tag and a Myc-tag (EQKLISEEDL). This construct is placed behind the l-arabinose inducible pBAD promoter. Constructs encoding variants of the enzymes were amplified from the corresponding plasmid using the mutagenic primers listed in Table 5. Following the amplification reaction, remaining template DNA was digested with DpnI and the mutated plasmids were transformed into DH5α *E. coli*. Subsequently, plasmid DNA was isolated and the introduction of the correct mutations was confirmed by sequencing. The mutated plasmids were transformed into BL21 (for His-VAO) or TOP10 (for EUGO-His) *E. coli* for protein expression.

### 4.3. Protein Expression and Purification

For the expression of His-VAO and its variants, BL21 *E. coli* containing the correct plasmid was grown in 100 mL LB medium (Duchefa Biochemie, Haarlem, The Netherlands) containing 100 μg/mL ampicillin at 37 °C until the OD_600_ was 0.6. Subsequently, protein expression was induced by adding IPTG to a final concentration of 0.8 mM and cells were grown overnight at 25 °C. Next, cells were harvested by centrifugation (4200× *g*, 15 min, 4 °C) and resuspended in 50 mM potassium phosphate buffer, pH 7.5, containing 500 mM NaCl, 5% glycerol (*v/v*), 0.5 mM MgSO_4_ and one cOmplete^TM^ protease inhibitor pill (Roche, Basel, Switzerland) and 1 mg DnaseI (Roche) per 50 mL. Cells were lysed by sonication using 6 cycles of 30 s at maximum power with an MSE sonication probe. Samples were cooled on ice during sonication. Following this, cell debris was removed by centrifugation (39,000× *g*, 45 min, 4 °C) and the supernatant was loaded onto a gravity flow column containing 2 mL HisPur Ni-NTA resin (Thermo Scientific, Waltham, MA, USA) equilibrated in 50 mM potassium phosphate buffer, pH 7.5, containing 500 mM NaCl and 5% glycerol. The column was washed with this buffer until all unbound proteins had eluted as judged from the absorption of the flow-through at 280 nm. Subsequently, His-VAO was eluted using 50 mM potassium phosphate buffer, pH 7.5, containing 500 mM NaCl, 5% glycerol (*v/v*) and 200 mM imidazole. Fractions containing His-VAO were pooled and transferred to 50 mM potassium phosphate buffer, pH 7.5, containing 150 mM NaCl and 10% glycerol by passing them over an Econo-Pac 10DG desalting column (Bio-Rad, Hercules, CA, USA) equilibrated in this buffer. This procedure typically yielded 1–3 mg protein from 100 mL *E. coli* culture.

After purification of F424G His-VAO, the protein did not contain any flavin cofactor. In an attempt to obtain flavin-containing protein, the purified F424G His-VAO variant was incubated with 1 mM FAD in 50 mM potassium phosphate buffer, pH 7.5, containing 75 mM NaCl and 5% glycerol at room temperature for 4 h. Subsequently, free FAD was removed by passing the mixture over an Econo-Pac 10DG desalting column (Bio-Rad) equilibrated in 50 mM potassium phosphate buffer, pH 7.5, containing 150 mM NaCl and 10% glycerol. Fractions containing the protein were collected and analysed for flavin content by measuring their absorption spectra. This revealed that the protein did not contain any FAD. No further attempts were made to incorporate FAD into the F424G His-VAO variant.

For the expression of EUGO-His and its variants, TOP10 *E. coli* containing the correct plasmid was grown in 100 mL TB medium (Difco, Becton Dickinson, Franklin Lake, NJ, USA) containing 100 μg/mL ampicillin at 37 °C until the OD_600_ was 0.6. Subsequently, protein expression was induced by adding l-arabinose to a final concentration of 0.02% (*w*/*v*) and cells were grown overnight at 30 °C. Next, cells were harvested by centrifugation (4200× *g*, 15 min, 4 °C) and resuspended in 50 mM potassium phosphate buffer, pH 7.5, containing 20 mM imidazole, 0.5 mM MgSO_4_ and one cOmplete^TM^ protease inhibitor pill (Roche) and 1 mg DnaseI (Roche) per 50 mL. Cells were lysed by sonication using 6 cycles of 30 s at maximum power with an MSE sonication probe. Samples were cooled on ice during sonication. Following this, cell debris was removed by centrifugation (39,000× *g*, 45 min, 4 °C) and the supernatant was loaded onto a gravity flow column containing 2 mL HisPur Ni-NTA resin (Thermo Scientific) equilibrated in 50 mM potassium phosphate buffer, pH 7.5, containing 20 mM imidazole. The column was washed with this buffer until all unbound proteins had eluted as judged from the absorption of the flow-through at 280 nm. Subsequently, EUGO-His was eluted using 50 mM potassium phosphate buffer, pH 7.5, containing 500 mM imidazole. Fractions containing EUGO-His were pooled and transferred into 50 mM potassium phosphate buffer, pH 7.5, by passing them over an an Econo-Pac 10DG desalting column (Bio-Rad) equilibrated in this buffer. This procedure typically yielded 5–10 mg protein from 100 mL *E. coli* culture.

### 4.4. Analytical Methods

All experiments were performed in 50 mM potassium phosphate buffer, pH 7.5, unless indicated otherwise. Absorption spectra were recorded on a Hewlett Packard 8453 photodiode array spectrophotometer (Agilent Technologies, Santa Clara, CA, USA). Protein concentrations of His-VAO and its variants were determined using the extinction coefficient of non-His-tagged VAO at 439 nm (ɛ_439_ = 12,500 M^−1^ cm^−1^ [3]) and concentrations of EUGO-His and its variants were determined using the extinction coefficient of non-His-tagged EUGO at 441 nm (ɛ_441_ = 14,200 M^−1^ cm^−1^ [4]). For trichloroacetic acid precipitations, 10 μL 50% (*w*/*v*) trichloroacetic acid solution was added to 90 μL 11 μM enzyme solution in 50 mM potassium phosphate buffer, pH 7.5, (EUGO-His and variants) or 50 mM potassium phosphate buffer, pH 7.5, containing 150 mM NaCl and 10% glycerol (His-VAO and variants) to yield final concentrations of 5% (*w*/*v*) trichloroacetic acid and 10 μM enzyme. Mixtures were incubated on ice for 30 min, after which the precipitate was pelleted by centrifugation (21,000× *g*, 15 min, 4 °C). The presence of flavin in the pellet or supernatant was judged from their colour and fluorescence upon irradiating them with UV-light. Steady-state kinetic parameters for the oxidation of vanillyl alcohol to vanillin by His-VAO and EUGO-His were determined by following the absorption of the product at 340 nm (ɛ_340_ = 14,000 M^−1^ cm^−1^) and fitting the obtained reaction rates to the Michaelis-Menten equation using IGOR Pro v. 6.10A (Wavemetrics, Lake Oswego, OR, USA). Oxygen consumption measurements were performed using a Hansatech Oxytherm system (Hansatech Instruments, King’s Lynn, UK).

### 4.5. Enzymatic Reactions for the Xylenol Orange Assay

Enzymatic reactions were performed in 96-wells plates. Substrate solution (180 µL) was added to 20 µL enzyme solution to give a reaction mixture containing 20 nM enzyme and 2 mM substrate in 50 mM potassium phosphate buffer, pH 7.5. For measurements where the oxidation of eugenol or vanillyl alcohol was followed in time, the reaction mixtures were incubated for the desired time (ranging from 1–20 min) at room temperature (19–20 °C), after which 20 μL of the reaction mixture was removed and the hydrogen peroxide concentration was determined using the xylenol orange assay as described below. Reaction rates were determined by fitting a curve to the linear range of the data using IgorPRO. For substrate specificity screening, the reaction mixtures were incubated for 10 min at room temperature (19–20 °C), after which a 20 μL sample was taken and the hydrogen peroxide concentration was determined using the xylenol orange assay as described below. Three of the tested compounds, 4-*n*-nonylphenol, 4-hydroxybenzyl alcohol and 4-cyclohexylphenol, were poorly soluble in water at the used concentration and therefore were added to the reaction mixtures as (partial) suspensions. Reaction rates were estimated by assuming that there is a linear increase in hydrogen peroxide concentration during the reaction time.

### 4.6. Xylenol Orange Assay

Twenty μL of the sample to be analysed was added to 180 μL xylenol orange assay mix in a 96-wells plate. This yielded a final assay mixture containing 100 μM xylenol orange, 250 μM ferrous sulphate, 25 mM sulfuric acid and the tenfold diluted analyte solution. This mixture was incubated in the dark at room temperature (19–20 °C) for 30 min, after which the absorption at 560 nm was measured using an xMark microplate spectrophotometer (Bio-Rad). A calibration curve of hydrogen peroxide solutions with known concentrations in the range 10–200 μM (concentration prior to addition to the xylenol orange assay mixture) was used to convert the measured absorbance values to the hydrogen peroxide concentration in the analyte solution. To ensure that the measured hydrogen peroxide concentrations reflect actual hydrogen peroxide formed during the enzymatic reaction, a detection limit of 10 μM hydrogen peroxide, which corresponds to the lowest point of the calibration curve, was used. All samples where the measured hydrogen peroxide concentration was lower than this were deemed to have had no significant formation of hydrogen peroxide during the reaction period.

### 4.7. Conversion of 4-cyclopentylphenol and 4-cyclohexylphenol and Identification of the Reaction Products

For GC and GC/MS analysis, reaction mixtures (100 μL) containing 2 mM substrate and 1 μM enzyme in 50 mM potassium phosphate buffer, pH 7.5, (EUGO-His) or 50 mM potassium phosphate buffer, pH 7.5, containing 30 mM NaCl and 2% (*w*/*v*) glycerol (His-VAO), were incubated for 2 h (4-cyclopentylphenol) or 4 h (4-cyclohexylphenol) at 25 °C under shaking (500 rpm). Subsequently, the reactions were stopped by extracting the reaction mixtures twice with 100 μL ethyl acetate. The ethyl acetate layers were combined and dried over Na_2_SO_4_. Reaction products were analysed by GC and GC/MS. GC was performed on an Agilent 6890 GC system equipped with an Agilent 7673 injector using a 60 m × 0.25 mm × 0.25 μm DB-1 column (Agilent Technologies). The injector temperature was 220 °C and the FID temperature was 250 °C. The split ratio was 10:1. Samples from reactions with 4-cyclopentylphenol were run at a flow rate of 1.7 mL/min using the following temperature programme: 100 °C for 5 min, followed by a temperature gradient to 230 °C with an increment of 10 °C per min, followed by 3 min at 230 °C. Samples from reactions with 4-cyclohexylphenol were run at a flow rate of 1.7 mL/min using the following temperature programme: 100 °C for 4 min, followed by a temperature gradient to 160 °C with an increment of 10 °C per min, followed by 10 min at 160 °C, followed by a temperature gradient to 230 °C with an increment of 10 °C per min, followed by 3 min at 230 °C. Reported conversion percentages are average values from duplicate experiments. GC/MS was performed on an Agilent 6890 GC system equipped with an Agilent 7975C MS detector and an Agilent 7683B injector using a 30 m × 0.25 mm × 0.25 μm HP-5MS column (Agilent Technologies). The injector temperature was 275 °C. The split ratio was 100:1. Samples were run at a flow rate of 1.1 mL/min using the following programme: 50 °C for 3 min, followed by a temperature gradient to 100 °C with an increment of 5 °C per min, followed by a temperature gradient to 250 °C with an increment of 10 °C per min, followed by 3 min at 250 °C.

For ^1^H-NMR analysis, a reaction mixture (25 mL) containing 2.5 mM 4-cyclopentylphenol and 0.5 μM EUGO-His was incubated in 50 mM potassium phosphate buffer, pH, 7.5, at 25 °C for 16 h under shaking (500 rpm). Following this, reaction products were extracted twice with 25 mL ethyl acetate. The ethyl acetate layers were combined and dried over Na_2_SO_4_. Subsequently, ethyl acetate was removed by evaporation and the remaining solid was dissolved in CDCl_3_. ^1^H-NMR spectra were recorded on an Avance III 400 MHz NMR spectrometer (Bruker, Billerica, MA, USA) at 400 MHz. This revealed that the obtained product was a mixture of 40% 4-cyclopentylphenol and 60% 4-(1-cyclopenten-1-yl)phenol. Chemical shifts (δ) are reported in parts per million (ppm) relative to CDCl_3_. Data is reported as follows: br = broad, s = singlet, d = doublet, m = multiplet, ap = apparent; coupling constant(s) (*J*) in Hz and integration.

*4-cyclopentylphenol.* ^1^H-NMR (400 MHz, CDCl_3_) δ 7.12 (ap d, *J* = 8.5 Hz, 2H), 6.77 (ap d, *J* = 8.5 Hz, 2H), 4.66 (s, 1H), 2.93 (m, 1H), 2.14–1.96 (m, 2H), 1.87–1.72 (m, 2H), 1.75–1.59 (m, 2H), 1.61–1.46 (m, 2H).

*4-(1-cyclopenten-1-yl)phenol*. ^1^H-NMR (400 MHz, CDCl_3_) δ 7.33 (ap d, *J* = 8.6 Hz, 2H), 6.78 (ap d, *J* = 8.9 Hz, 2H), 6.04 (m, 1H), 4.80 (br s, 1H), 2.73–2.56 (m, 2H), 2.56–2.45 (m, 2H), 2.09–1.96 (m, 2H).

## Figures and Tables

**Table 1:** Steady-state kinetic parameters for the oxidation of vanillyl alcohol by vanillyl alcohol oxidase (VAO), His-tagged vanillyl alcohol oxidase (His-VAO), eugenol oxidase (EUGO) and His-tagged eugenol oxidase (EUGO-His) in 50 mM potassium phosphate buffer, pH 7.5, at 25 °C.

Enzyme	*k*_cat_ (s^−1^)	*K*_m_ (μM)	*k*_cat_/*K*_m_ (s^−1^ mM^−1^)
VAO	2.6 ^a^	149 ^a^	17
His-VAO	2.5 ± 0.1	129 ± 15	19
EUGO	7.1 ^a^	79 ^a^	90
EUGO-His	8.0 ± 0.2	47 ± 5	170

^a^ Data from [13].

**Table 2:** Reaction rates for the oxidation of 2 mM vanillyl alcohol or eugenol by His-tagged vanillyl alcohol oxidase (His-VAO) or His-tagged eugenol oxidase (EUGO-His) in 50 mM potassium phosphate buffer, pH 7.5, at room temperature (19–20 °C) as determined using the xylenol orange assay. The *k*_cat_ values for the non-His-tagged enzymes measured previously using other methods are shown for comparison. These *k*_cat_ values were measured under identical experimental conditions, with the exception of the temperature, which was 25 °C instead of 19–20 °C.

	(His)-VAO		EUGO-(His)	
	Vanillyl-alcohol	Eugenol	Vanillyl-alcohol	Eugenol
*k*_obs_ Xylenol Orange Assay (s^−1^)	1.5 ± 0.1	8.0 ± 0.3	6.6 ± 0.1	3.2 ± 0.1
*k*_cat_ (s^−1^)	2.6 ^a^	11^a^	7.1 ^a^	2.9 ^a^

^a^ Data taken from [13].

**Table 3:** Compounds tested in the substrate specificity screening.

Entry	Compound Name (Trivial Name)	Structure
**1**	4-methylphenol (*para*-cresol)	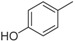
**2**	2-methoxy-4-methylphenol (*para*-creosol)	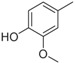
**3**	4-ethylphenol	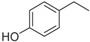
**4**	4-*n*-propylphenol	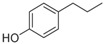
**5**	4-isopropyphenol	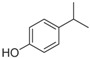
**6**	4-*n*-butylphenol	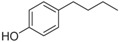
**7**	4-*sec*-butylphenol	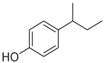
**8**	4-*n*-pentylphenol	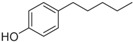
**9**	4-*n*-hexylphenol	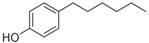
**10**	4-*n*-nonylphenol	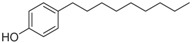
**11**	2-hydroxybenzyl alcohol	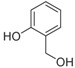
**12**	3-hydroxybenzyl alcohol	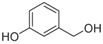
**13**	4-hydroxybenzyl alcohol	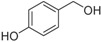
**14**	4-hydroxy-3-methoxybenzyl alcohol (vanillyl alcohol)	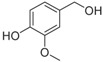
**15**	4-hydroxy-3,5-dimethoxybenzyl alcohol	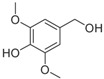
**16**	4-hydroxy-2-methoxybenzyl alcohol	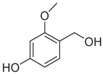
**17**	4-allylphenol (chavicol)	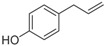
**18**	4-allyl-2-methoxyphenol (eugenol)	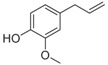
**19**	4-allyl-2,6-dimethoxyphenol	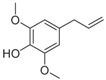
**20**	5-indanol	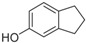
**21**	5,6,7,8-tetrahydro-2-naphthol	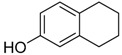
**22**	4-cyclopentylphenol	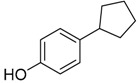
**23**	4-cyclohexylphenol	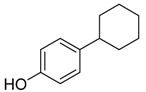
**24**	4-(aminomethyl)-2-methoxyphenol (vanillyl amine)	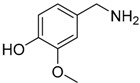

**Table 4:** List of residues forming part of the substrate-binding pocket that are different in vanillyl alcohol oxidase (VAO) and eugenol oxidase (EUGO) and of the His-tagged vanillyl alcohol oxidase (His-VAO) and His-tagged eugenol oxidase (EUGO-His) variants that were made during this study. The residues are numbered according to their position in the non-His-tagged wild-type proteins.

Position	Residue in His-VAO	Residue in EUGO-His	His-VAO Variant	EUGO-His Variant
1	Leu-316	Met-282	L316M	M282L
2	Trp-413	Leu-381	W413L	L381W
3	Phe-424	Gly-392	F424G	G392F
4	Thr-457	Gln-425	T457Q	Q425T
5	Thr-459	Ile-427	T459I	I427T
6	Ile-468	Val-436	I468V	V436I
7	Cys-470	Leu-438	C470L	L438C

**Table 5:** List of primers used for site-directed mutagenesis in this study. Sites in italics indicate the position of the mutations introduced to achieve the amino acid substitution. Underlined sites indicate the position of mutations introduced to remove or introduce a restriction site to enable screening for the introduction of the correct mutations by restriction enzyme digestion (these are either the same mutations used for the amino acid substitution or silent mutations close to this site). The identity of the used restriction enzymes is given in the third column (+ indicates that a restriction site for this enzyme was introduced, − indicates that a restriction site for this enzyme was removed). His-VAO: His-tagged vanillyl alcohol oxidase, EUGO-His: His-tagged eugenol oxidase.

Variant	Primer	Restriction Site
L316M His-VAO	ATCCGCCATATTCTG*A*TGGATGCTGCAGTGCTGGGTGACAAG	+PstI
W413L His-VAO	TATGATGAACTGAAG*CTT*ATTGATTGGTTGCCG	+HindIII
F424G His-VAO	AATGGTGCCCACCTG*GGT*TTCAGCCCGATTGCGAAGGTGTCTGGT	−PvuI
T457Q His-VAO	TGCCAGGAAGCAGGTCTAGACTTCATTGGT*CAA*TTCACTGTTGGTATG	+XbaI
T459I His-VAO	TTCATTGGTACGTTCA*T*TGTTGGTATGCGTGAG	None
I468V His-VAO	ATGCGTGAGATGCATCAT*G*TTGTTTGCATCGTGTTT	+NsiI
C470L His-VAO	ATGCGTGAGATGCATCATATTGTTT*TA*ATCGTGTTTAACAAG	+NsiI
M282L EUGO-His	CTGCGCAACATCTTC*T*TGGACGCGGCCGCGGTG	None
L381W EUGO-His	CTGGACGAACTGCAG*TGG*CTGGACTGGGTGCCC	+PstI
G392F EUGO-His	AACGGCGGGCACATC*TTT*TTCTCACCGGTGTCGGCGCCG	+AgeI
Q425T EUGO-His	AAGGACTACGCCGCG*ACA*TTCATCATCGGGCTC	None
I427T EUGO-His	TACGCCGCGCAATTC*ACG*ATCGGGCTCCGCGAG	+PvuI
V436I EUGO-His	CGCGAGATGCACCAC*A*T*A*TGCCTGTTCATCTAC	+NdeI
L438C EUGO-His	CTCCGCGAGATGCATCACGTGTGC*TGC*TTCATCTACGACACG	+NsiI

## Supplementary Materials

The following are available online. Figure S1: Absorption spectra of His-VAO and its variants, Figure S2: Absorption spectra of EUGO-His and its variants, Figure S3: GC chromatograms of the reaction products of the conversion of 4-cyclopentylphenol by His-VAO and EUGO-His; Figure S4: GC/MS chromatograms and mass spectra of the reaction products of the conversion of 4-cyclopentylphenol by His-VAO and EUGO-His, Figure S5: ^1^H-NMR spectra of 4-cyclopentylphenol and the reaction products of its conversion by EUGO-His, Figure S6: GC chromatograms of the reaction products of the conversion of 4-cyclohexylphenol by His-VAO, EUGO-His and W413L His-VAO, Figure S7: GC/MS chromatograms and mass spectra of the reaction products of the conversion of 4-cyclohexylphenol by His-VAO, EUGO-His and W413L His-VAO.
