# Supplementary material for: A Xylenol Orange-Based Screening Assay for the Substrate Specificity of Flavin-Dependent para-Phenol Oxidases
Source: Molecules. 2018 Jan 14;23(1):164. doi: 10.3390/molecules23010164 (PMC6017454; doi:10.3390/molecules23010164)
Supplement: Supplementary file 1 [file molecules-23-00164-s001.pdf]

Supplementary Information

A xylenol orange-based screening assay for the substrate specificity of flavin-dependent *para*-phenol oxidases

Tom A. Ewing, Aster van Noord, Caroline E. Paul, Willem J. H. van Berkel

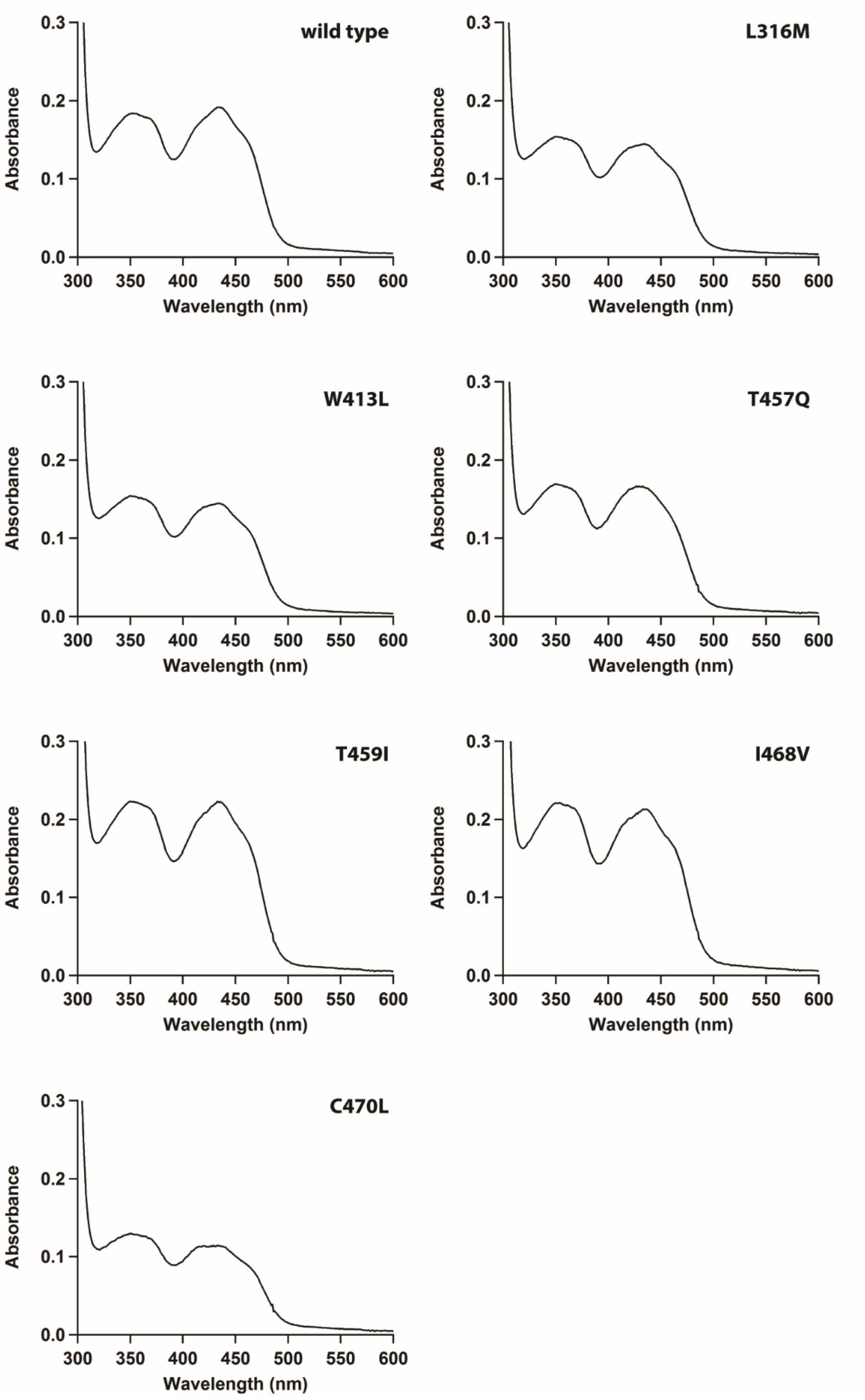

Figure S1: Absorption spectra of His-VAO variants recorded in 50 mM potassium phosphate buffer, pH 7.5, at 25 °C.

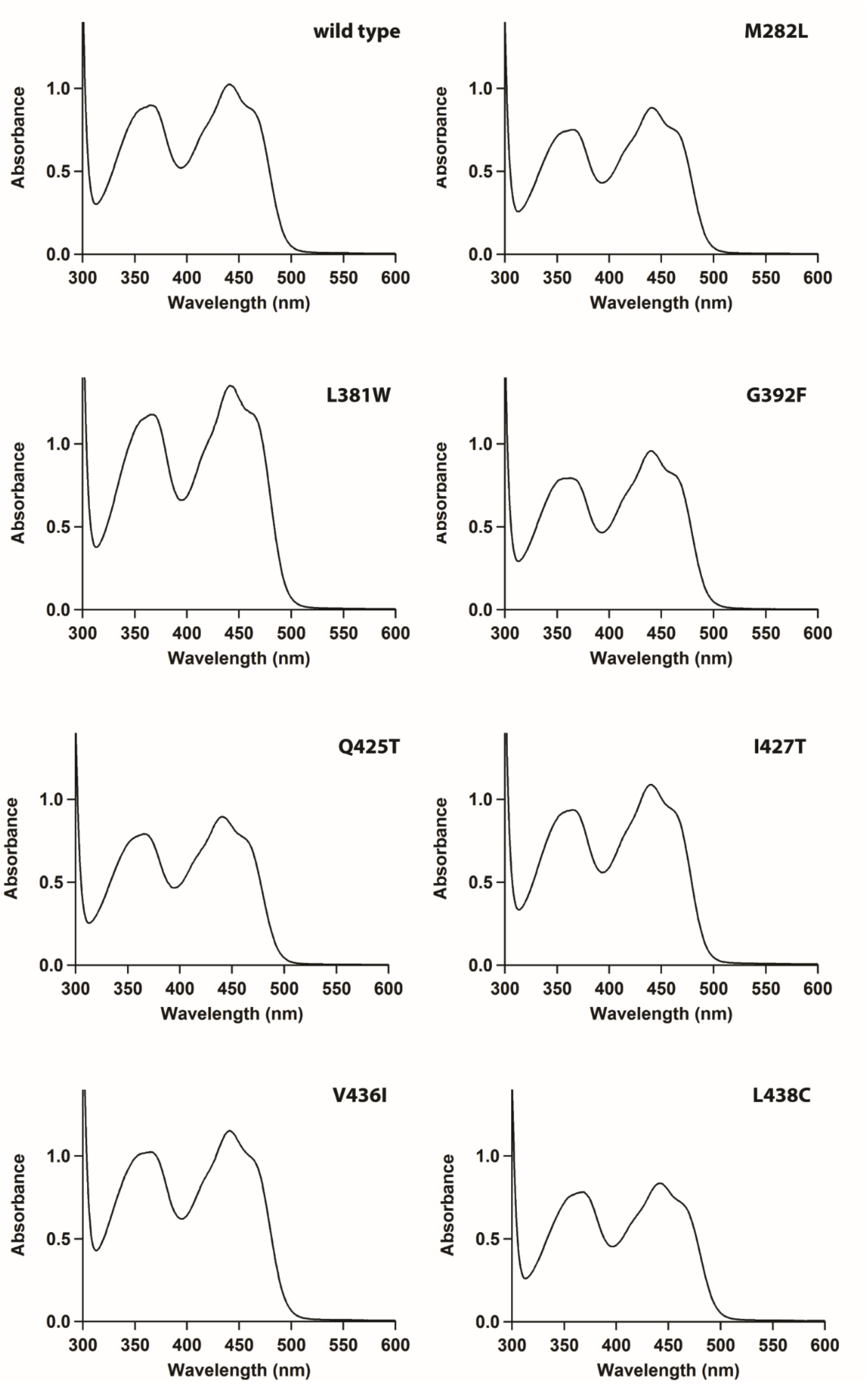

Figure S2: Absorption spectra of EUGO-His variants recorded in 50 mM potassium phosphate buffer, pH 7.5, at 25 °C.

4-cyclopentylphenol

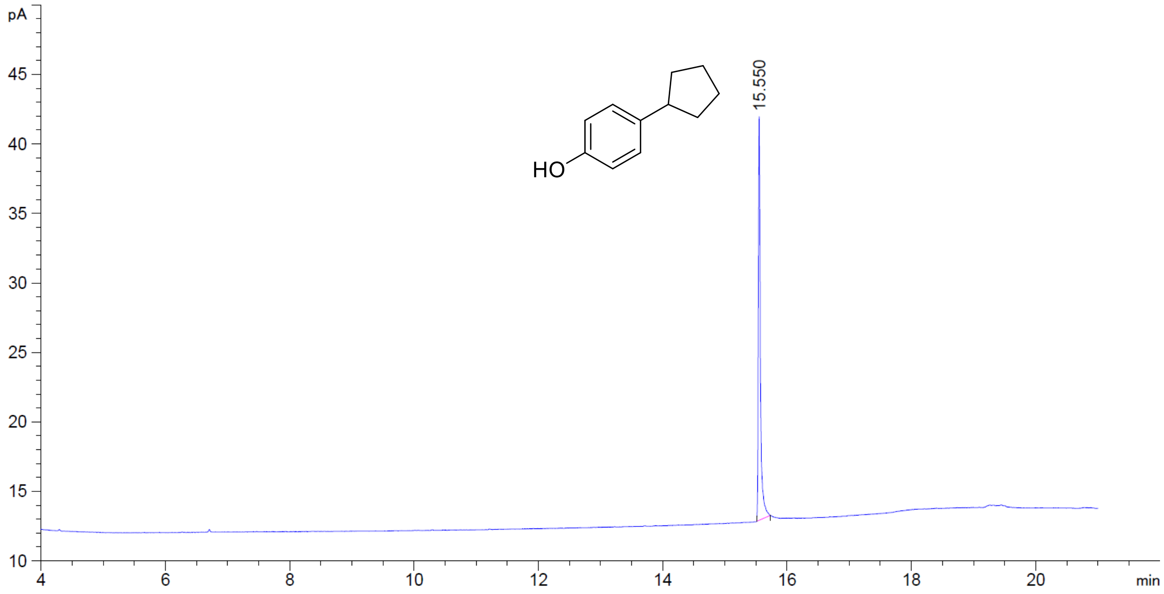

His-VAO reaction

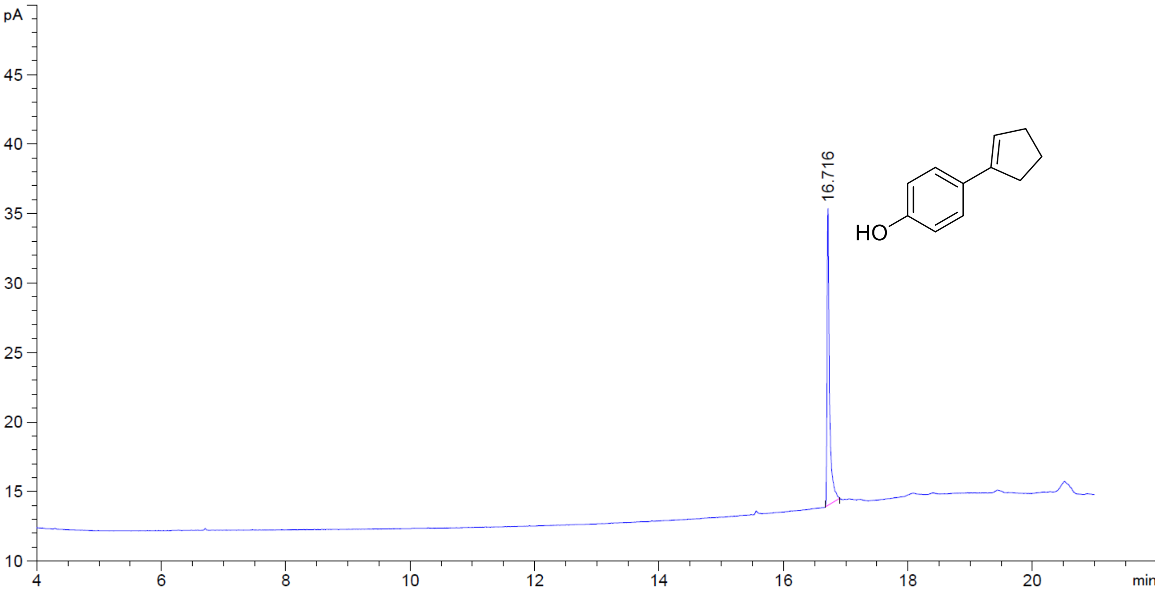

EUGO-His reaction

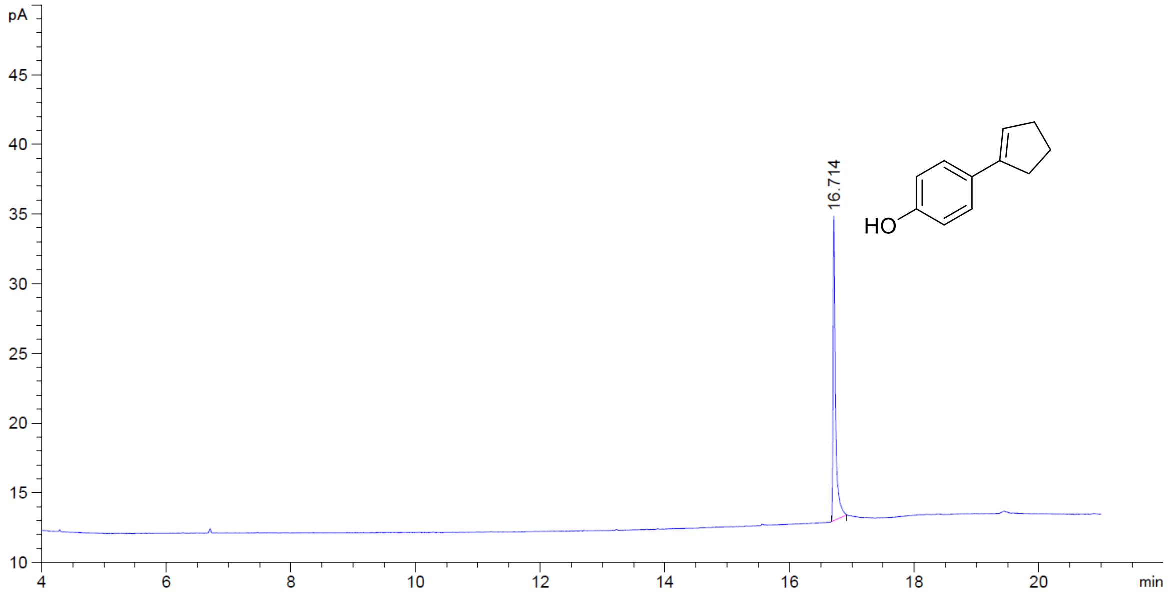

Figure S3: GC chromatograms of the reaction products of the conversion of 4-cyclopentylphenol by His-VAO or EUGO-His. 2 mM 4-cyclopentylphenol and 1 µM enzyme were incubated for 2 h at 25 °C in 50 mM potassium phosphate buffer, pH 7.5, (EUGO-His) or 50 mM potassium phosphate buffer, pH 7.5, containing 30 mM NaCl and 2% (w/v) glycerol (His-VAO). Subsequently, the reaction products were extracted and analysed by GC. For both reactions, a single product, 4-(1-cyclopenten-1-yl)phenol, was formed at >99% conversion. Retention times: 4-cyclopentylphenol, 15.6 min; 4-(1-cyclopenten-1-yl)phenol, 16.7 min.

4-cyclopentylphenol

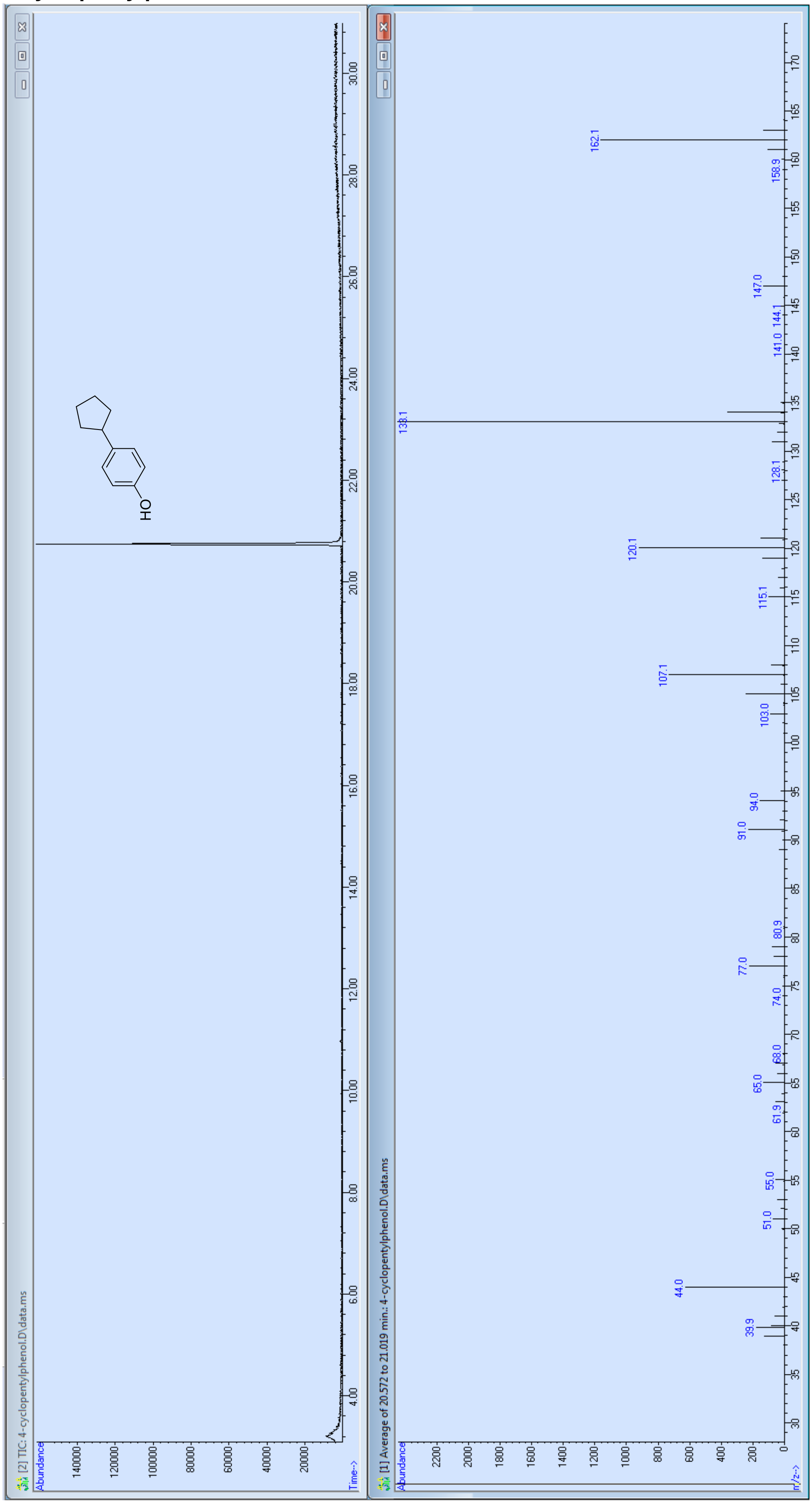

His-VAO reaction

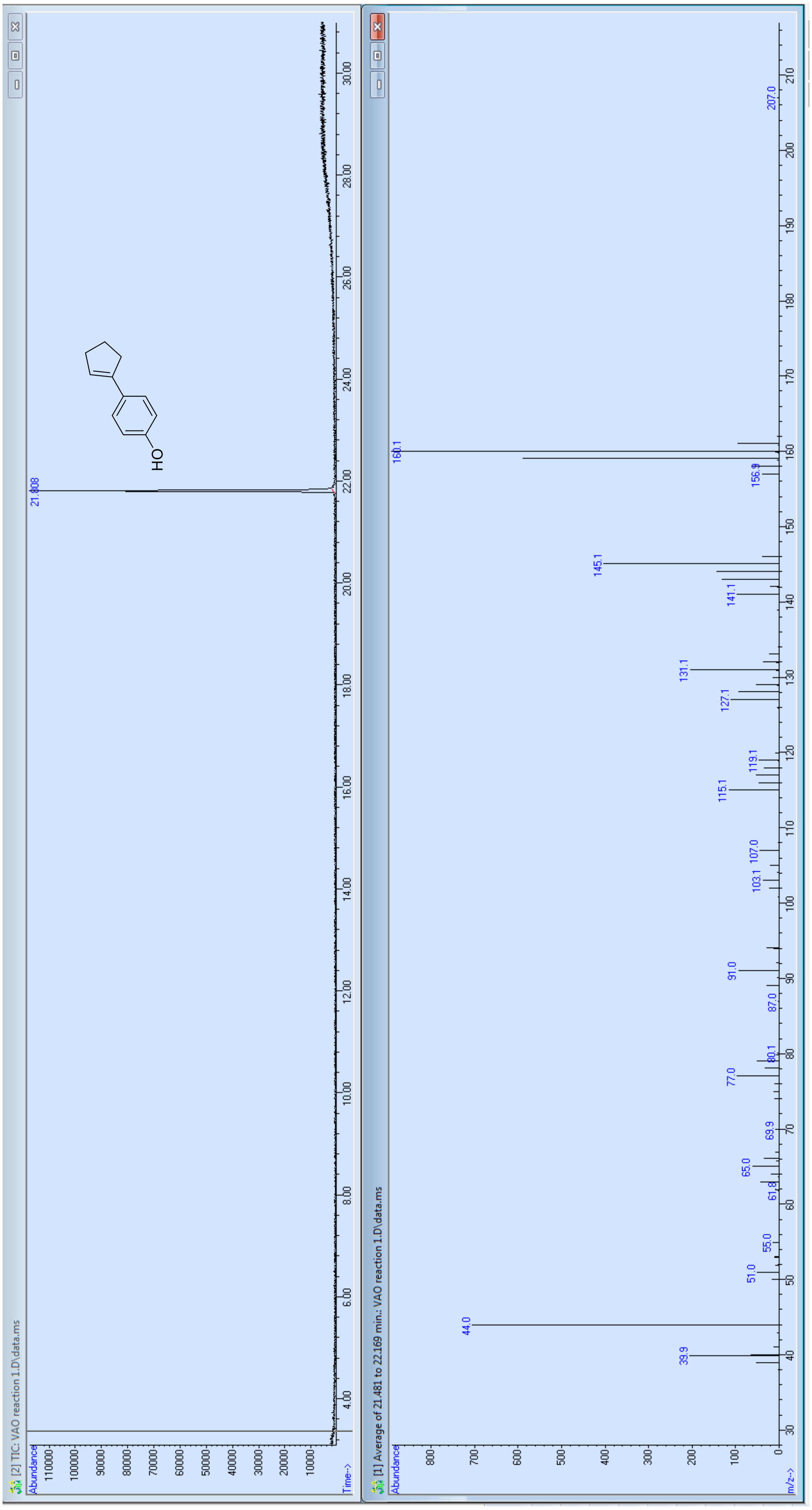

EUGO-His reaction

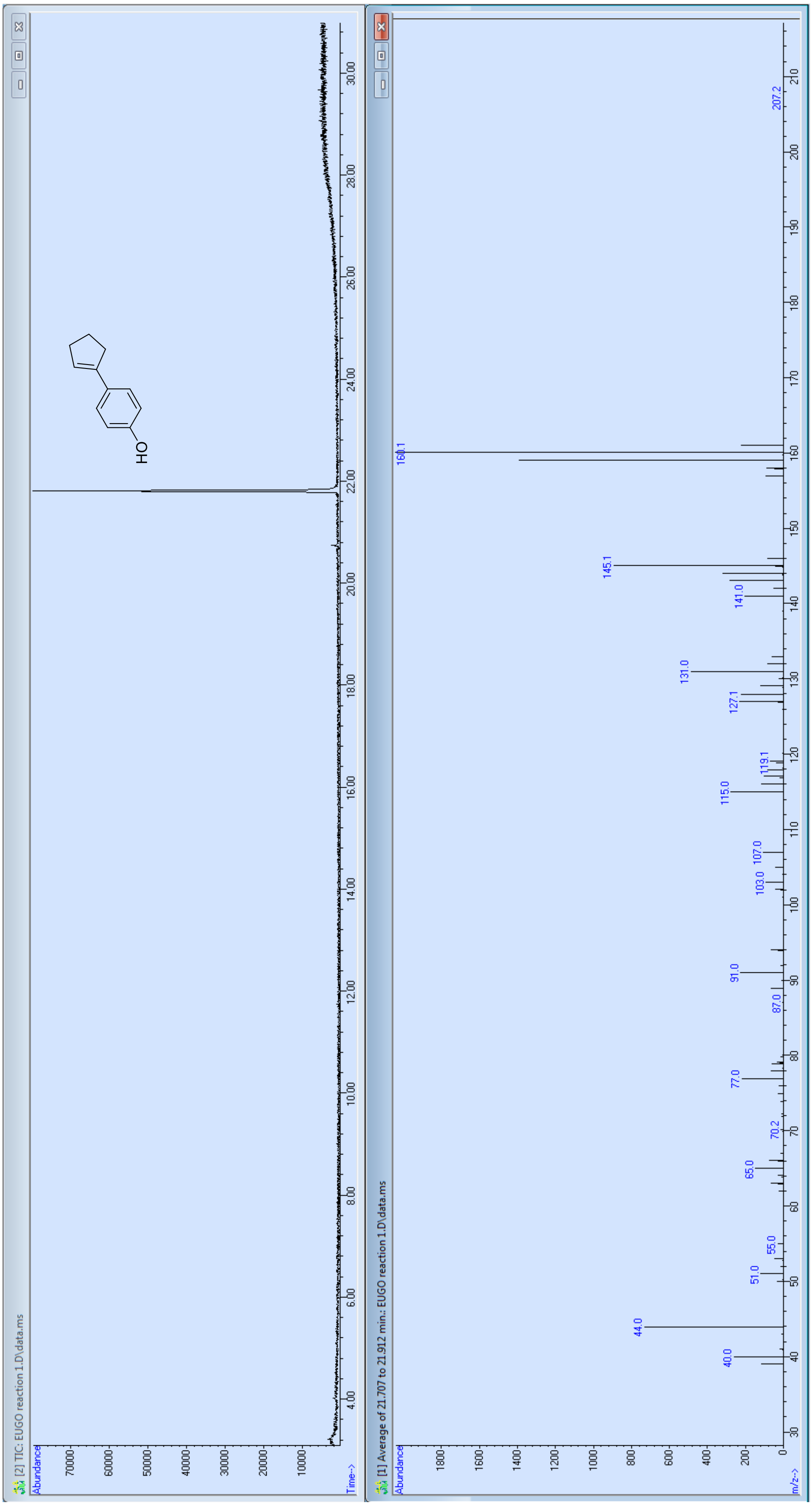

Figure S4: GC/MS analysis of the reaction products of the conversion of 4-cyclopentylphenol by His-VAO or EUGO-His. 2 mM 4-cyclopentylphenol and 1 μM enzyme were incubated for 2 h at 25 °C in 50 mM potassium phosphate buffer, pH 7.5, (EUGO-His) or 50 mM potassium phosphate buffer, pH 7.5, containing 30 mM NaCl and 2% (w/v) glycerol (His-VAO). Subsequently, the reaction products were extracted and analysed by GC/MS. For both reactions, a single product, 4-(1-cyclopenten-1-yl)phenol, was formed. Top panels show GC chromatograms with retention times: 4-cyclopentylphenol, 21.0 min; 4-(1-cyclopenten-1-yl)phenol, 21.8 min. Bottom panels show mass spectra recorded of the eluting compounds.

4-cyclopentylphenol

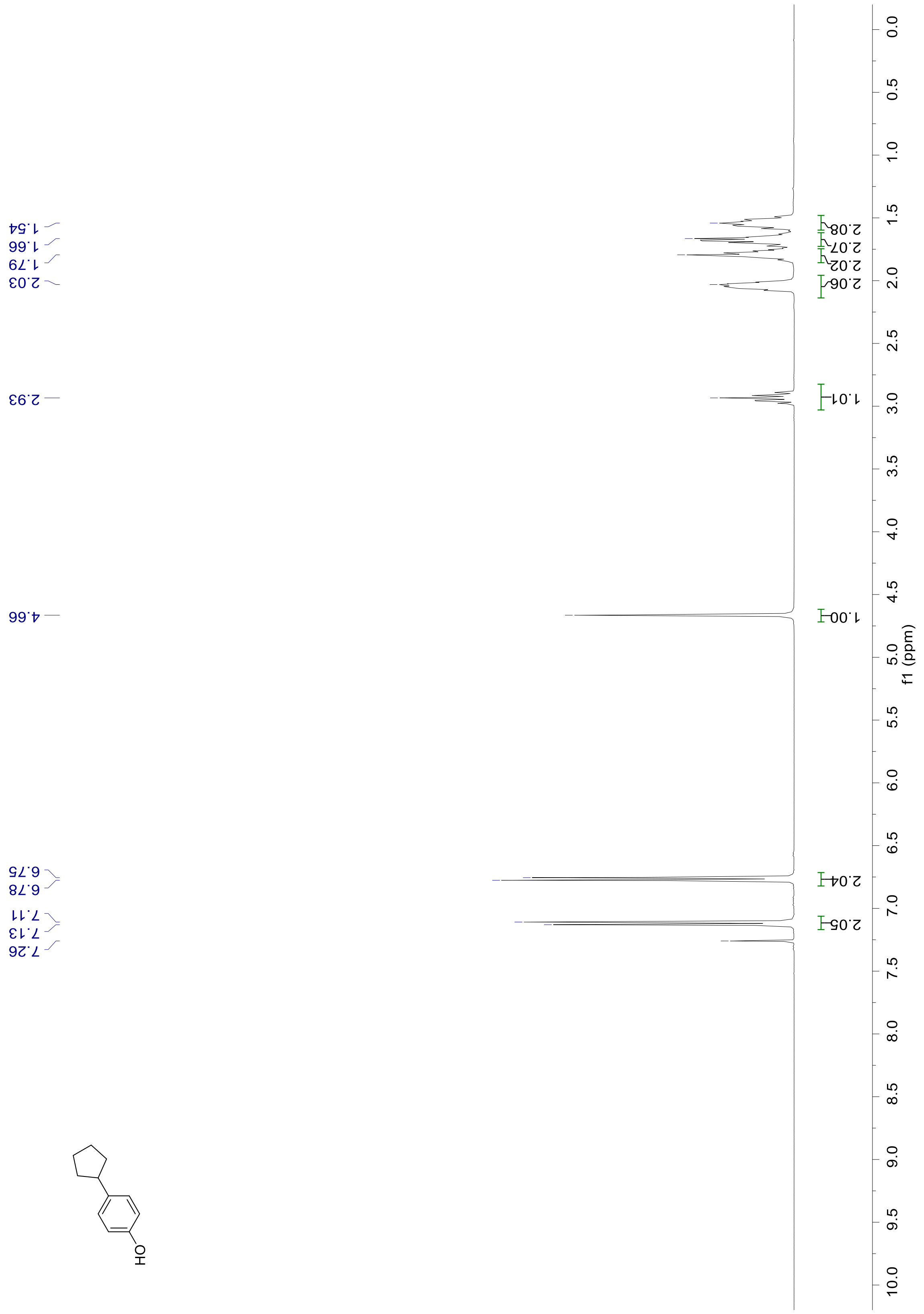

EUGO-His reaction

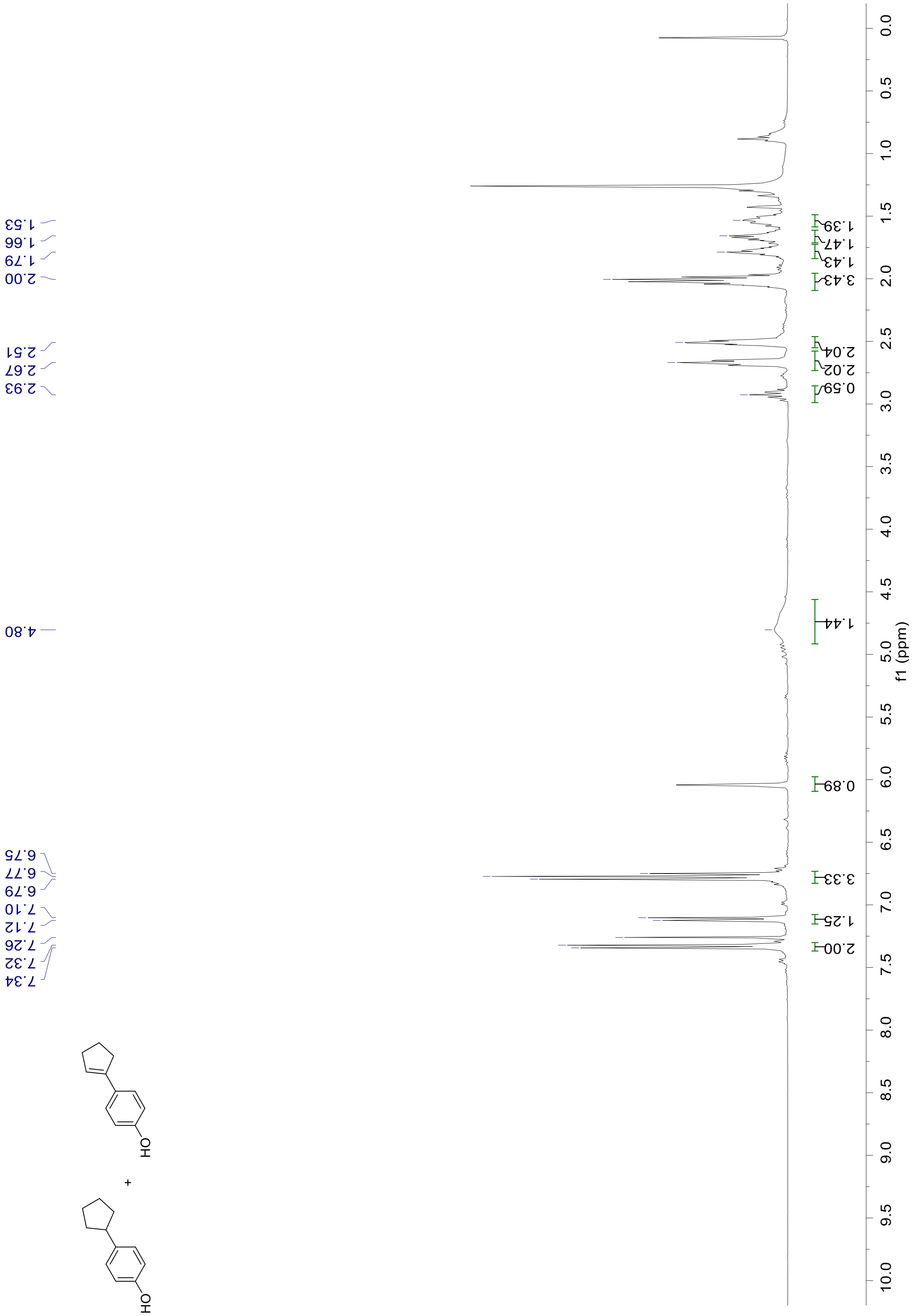

Figure S5: ^{1}H-NMR spectra of 4-cyclopentylphenol and the products of the conversion of 4-cyclopentylphenol by EUGO-His. 2.5 mM 4-cyclopentylphenol and 0.5 μM EUGO-His were incubated in 50 mM potassium phosphate buffer, pH 7.5, at 25 °C for 16 h. Subsequently, reaction products were extracted and analysed by ^{1}H-NMR. This revealed that the product is a mixture of 4-cyclopentylphenol (40%) and 4-(1-cyclopenten-1-yl)phenol (60%). Chemical shifts (δ) are reported in parts per million (ppm) relative to $CDCl_3$. Data is reported as follows: br = broad, s = singlet, d = doublet, m = multiplet, ap = apparent; coupling constant(s) (J) in Hz and integration. Peaks below 1.5 ppm are due to impurities in the $CDCl_3$. **4-cyclopentylphenol**: ^{1}H-NMR (400 MHz, $CDCl_3$) δ 7.12 (ap d, J = 8.5 Hz, 2H), 6.77 (ap d, J = 8.5 Hz, 2H), 4.66 (s, 1H), 2.93 (m, 1H), 2.14 – 1.96 (m, 2H), 1.87 – 1.72 (m, 2H), 1.75 – 1.59 (m, 2H), 1.61 – 1.46 (m, 2H). **4-(1-cyclopenten-1-yl)phenol**: ^{1}H-NMR (400 MHz, $CDCl_3$) δ 7.33 (ap d, J = 8.6 Hz, 2H), 6.78 (ap d, J = 8.9 Hz, 2H), 6.04 (m, 1H), 4.80 (br s, 1H), 2.73 – 2.56 (m, 2H), 2.56 – 2.45 (m, 2H), 2.09 – 1.96 (m, 2H).

4-cyclohexylphenol

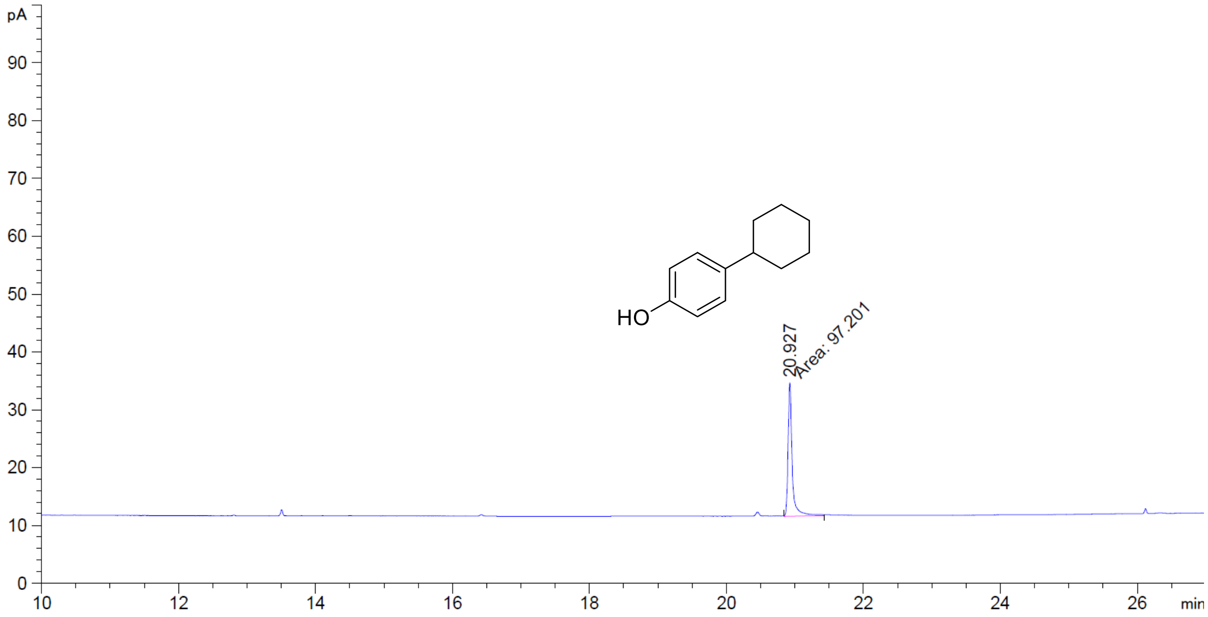

His-VAO reaction

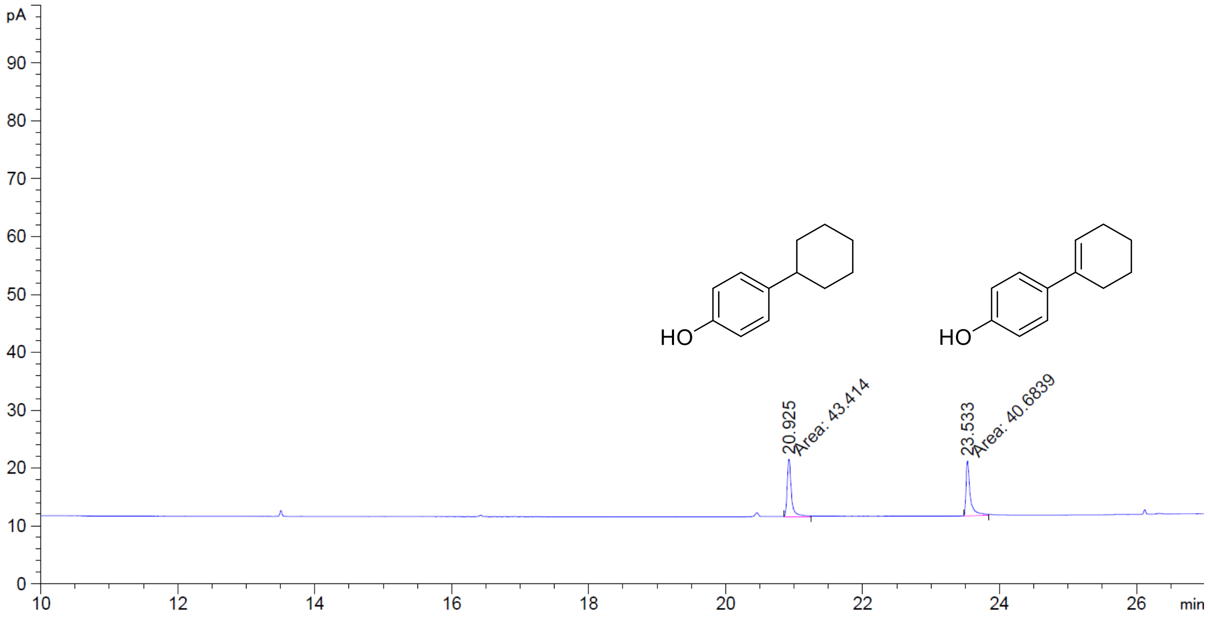

EUGO-His reaction

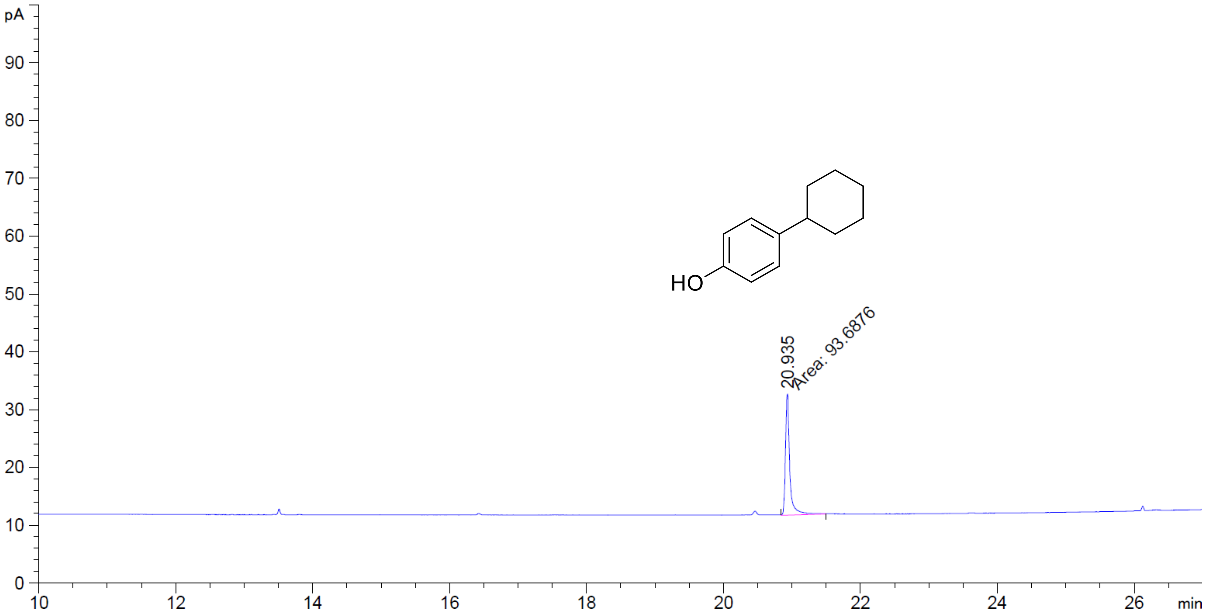

W413L His-VAO reaction

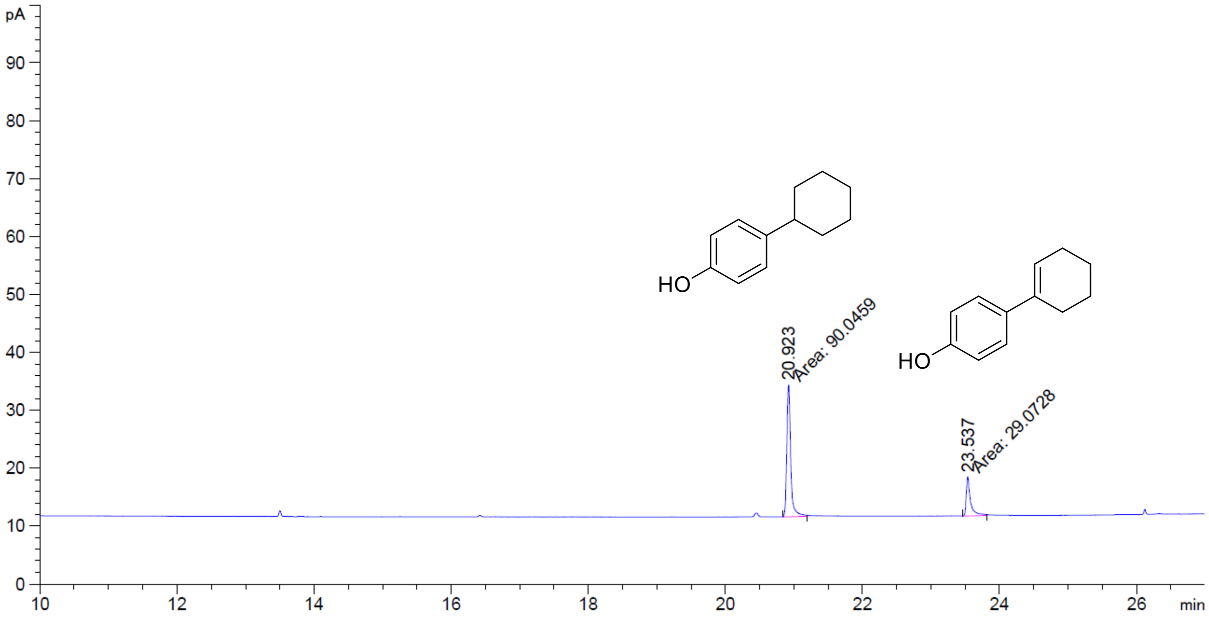

Figure S6: GC chromatograms of the reaction products of the conversion of 4-cyclohexylphenol by His-VAO, EUGO-His or W413L His-VAO. 2 mM 4-cyclohexylphenol and 1 µM enzyme were incubated for 4 h at 25 °C in 50 mM potassium phosphate buffer, pH 7.5, (EUGO-His) or 50 mM potassium phosphate buffer, pH 7.5, containing 30 mM NaCl and 2% (w/v) glycerol (His-VAO and W413L His-VAO). Subsequently, the reaction products were extracted and analysed by GC. With His-VAO and W413L His-VAO, a single product, 4-(1-cyclohexen-1-yl)phenol, was formed at 51% and 27% conversion respectively (average of two reactions). With EUGO-His, no conversion of the substrate was observed. Retention times: 4-cyclohexylphenol, 20.9 min; 4-(1-cyclohexen-1-yl)phenol, 23.5 min.

4-cyclohexylphenol

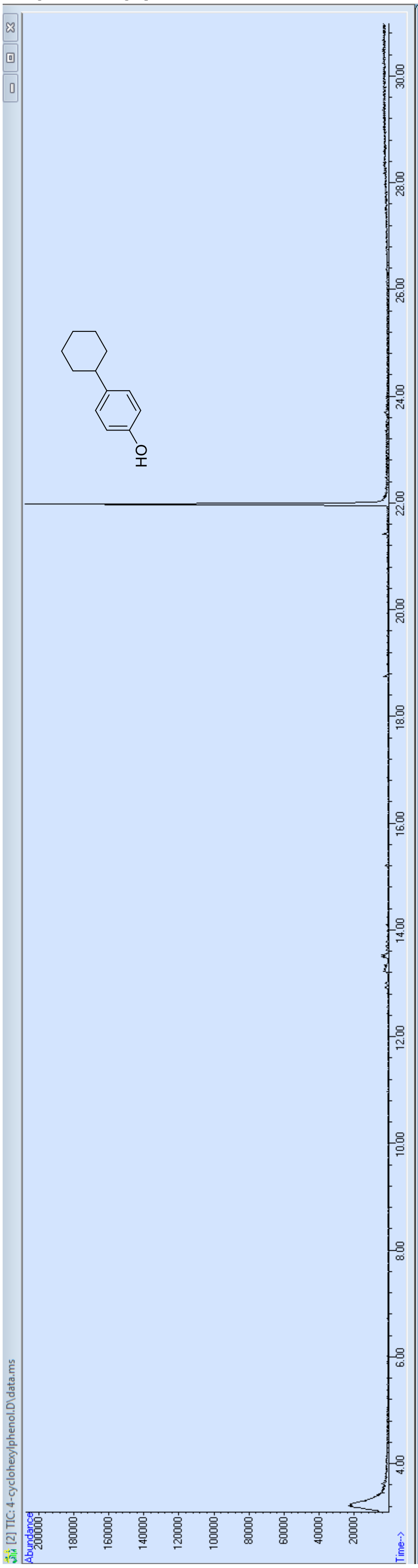

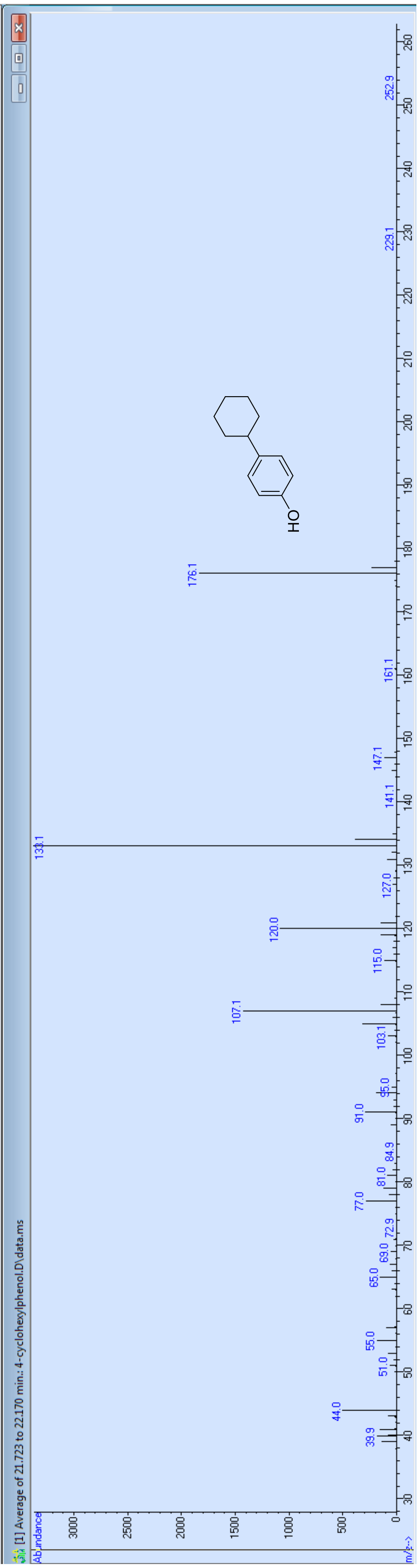

His-VAO reaction

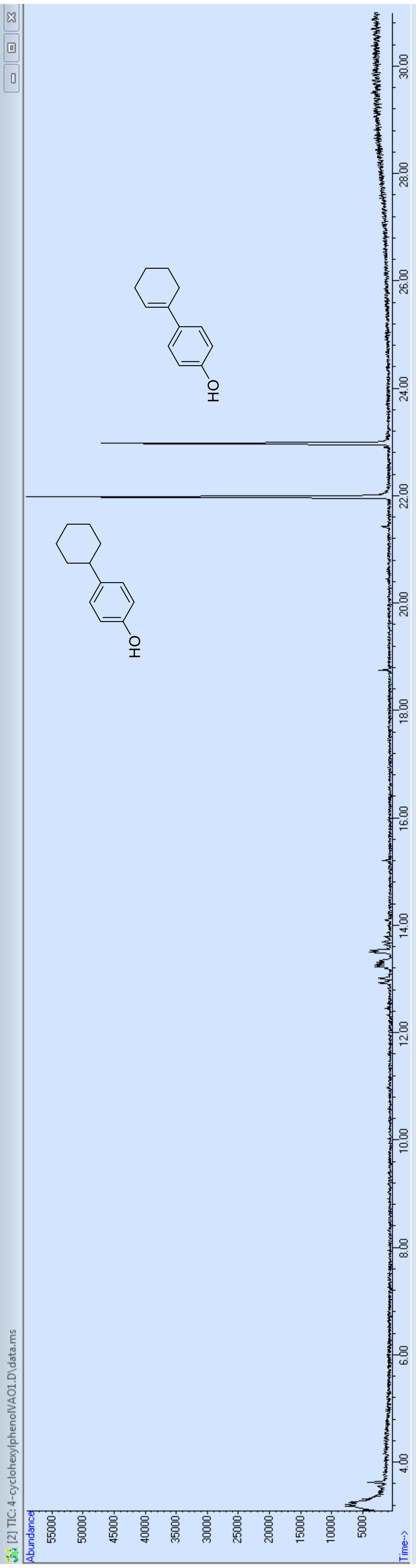

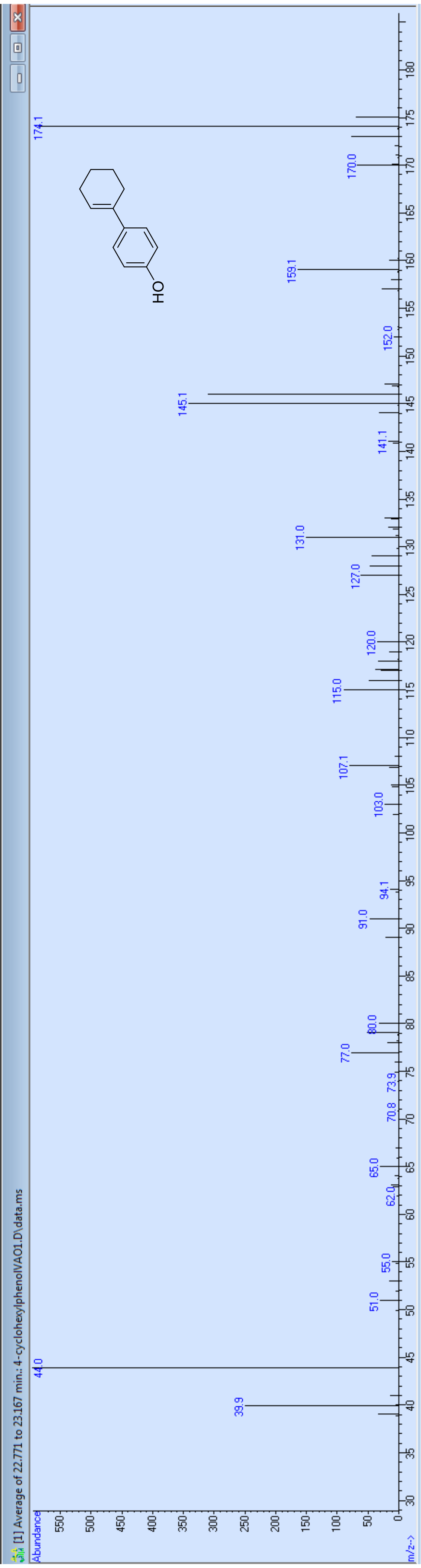

W413L His-VAO reaction

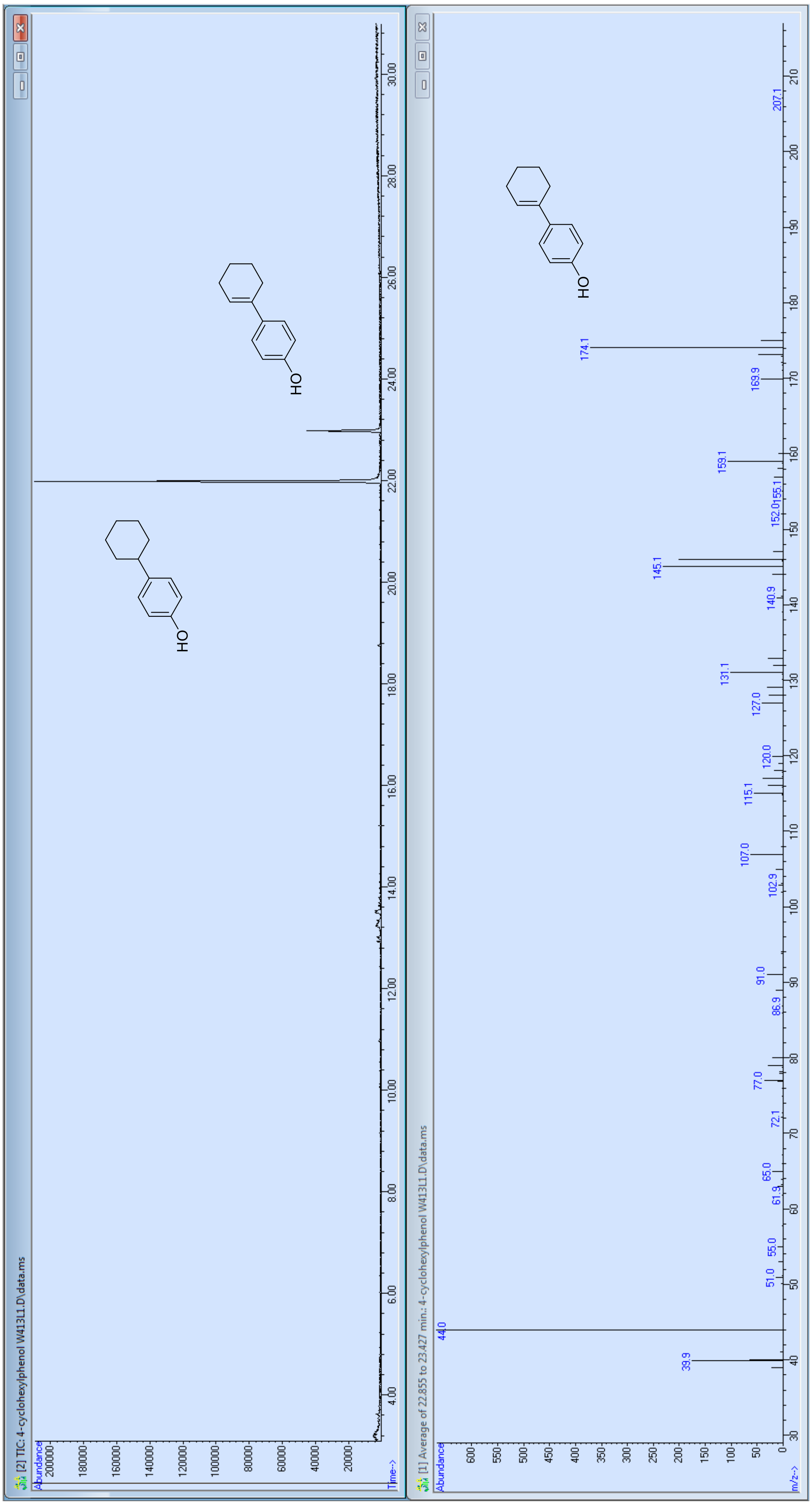

Figure S7: GC/MS analysis of the reaction products of the conversion of 4-cyclohexylphenol by His-VAO or W413L His-VAO. 2 mM 4-cyclohexylphenol and 1 µM enzyme were incubated for 4 h at 25 °C in 50 mM potassium phosphate buffer, pH 7.5, containing 30 mM NaCl and 2% (w/v) glycerol. Subsequently, the reaction products were extracted and analysed by GC/MS. For both reactions, a single product, 4-(1-cyclohexen-1-yl)phenol, was formed. Top panels show GC chromatograms with retention times: 4-cyclohexylphenol, 22.0 min; 4-(1-cyclohexen-1-yl)phenol, 23.0 min. Bottom panels show mass spectra recorded of the eluting compounds.
